# Pilot study of a multi-pronged intervention using social norms and priming to improve adherence to antiretroviral therapy and retention in care among adults living with HIV in Tanzania

**DOI:** 10.1371/journal.pone.0177394

**Published:** 2017-05-09

**Authors:** Sandra I. McCoy, Carolyn Fahey, Aarthi Rao, Ntuli Kapologwe, Prosper F. Njau, Sergio Bautista-Arredondo

**Affiliations:** 1 Division of Epidemiology, University of California, Berkeley, California, United States of America; 2 Enterprise Product Innovation, CVS Health, Rhode Island, United States of America; 3 Regional Medical Office, Ministry of Health, Community Development, Gender, Elderly, and Children, Shinyanga, Tanzania; 4 Prevention of Mother-to-Child HIV Transmission Programme, Ministry of Health, Community Development, Gender, Elderly, and Children, Dar es Salaam, Tanzania; 5 Health Economics Department, National Institute of Public Health, Cuernavaca, Mexico; Johns Hopkins University, UNITED STATES

## Abstract

**Background:**

Interventions incorporating constructs from behavioral economics and psychology have the potential to enhance HIV ‘treatment as prevention’ (TasP) strategies. To test this hypothesis, we evaluated an intervention to improve antiretroviral therapy (ART) adherence based on the concepts of social norms and priming.

**Methods:**

We used tools from marketing research and patient-centered design to develop a combination intervention that included visual feedback about clinic-level retention in care, a self-relevant prime, and useful take-home items with the priming image. The intervention was implemented at two HIV primary clinics in Shinyanga, Tanzania in 2-week intervals for six months. We conducted a quasi-experimental pilot study with a random sample of exposed and unexposed adult patients living with HIV infection (PLHIV) to compare retention and the proportion of patients with medication possession ratio (MPR) ≥95% after six months. Intervention acceptability was determined with a convenience sample of 405 PLHIV at baseline (n = 189) and endline (n = 216).

**Results:**

Medical records were reviewed for 438 PLHIV (320 intervention, 118 standard of care). In adjusted analyses, PLHIV exposed to the intervention were significantly more likely to be in care after 6 months (87% vs. 79%, adjusted odds ratio (OR_a_) = 1.73, 95% CI: 1.08, 2.78, p<0.05) and were more likely to achieve MPR≥95% (70% vs. 59%, OR = 1.51, 95% CI: 0.96, 2.37, p = 0.07). The intervention was associated with increases in staff support of treatment goals (100% vs. 95%, p = 0.01) and life goals (66% vs. 50%, p<0.01), the perceived likelihood of other patients’ adherence (54% vs. 32%, p<0.01), support from other patients (71% vs. 60%, p = 0.03), and being very satisfied with care (53% vs. 35%, p<0.01).

**Conclusions:**

This novel intervention has the potential to improve the clinic experience, short-term retention in care, and ART adherence. Future studies are needed to expand the generalizability of the approach and evaluate effectiveness on clinical outcomes.

**Trial registration:**

Clinicaltrials.gov NCT02938533

## Introduction

Most antiretroviral therapy (ART) regimens require more than 80–95% adherence to maximize the probability of viral suppression,[1–4] the ultimate goal of treatment that has significant clinical benefits and can virtually eliminate HIV transmission.[5, 6] However, in sub-Saharan Africa nearly 25% of people living with HIV infection (PLHIV) on treatment have sub-optimal adherence and overall, only 29% of PLHIV are virally suppressed.[7, 8] Furthermore, disengagement from care is a pervasive threat to the effectiveness of ‘treatment as prevention’ (TasP) strategies,[5] as only 65% of PLHIV in Africa are retained in care 36 months after treatment initiation.[9] Thus, the common goal of governments and donors to reduce HIV incidence and extend the lives of PLHIV necessitates creative, effective, and scalable strategies to retain PLHIV in care and bolster ART adherence.[10]

Traditional strategies to improve ART treatment adherence, and health in general, often erroneously assume that information is sufficient to change behavior. Such interventions primarily rely on individuals’ innate desire to remain healthy; however, they ignore that decisions are influenced by emotions, contexts, and systems as well as decision-making shortcuts that are often outside of conscious awareness.[11] In contrast, there is growing interest in interventions incorporating constructs from behavioral economics and psychology to account for our predicable irrationalities, heuristics, and biases–thereby changing behavior without relying solely on intrinsic or extrinsic motivation.[11–15] These strategies encompass approaches such as incentives, defaults, and commitments, and they have been increasingly utilized to promote weight loss, smoking cessation, and the adoption of other beneficial health behaviors.[11, 15] However, there have been few applications to HIV/AIDS programs with the exception of cash and in-kind incentives.[16, 17]

Using tools from marketing research and patient-centered design,[18] we developed and implemented an intervention to improve ART adherence based on the concepts of *norms* and *priming*. Social norms and social influence are increasingly recognized as powerful behavior change tools.[11, 19] For example, a recent study reduced inappropriate antibiotic prescribing by informing frequent prescribers that they were prescribing at a higher rate than 80% of other practices.[20] After the intervention, there was a 3.3% reduction in antibiotic prescribing, translating to 73,406 fewer prescriptions during the short 6-month study period.[20] Programs targeting social norms by engaging opinion leaders have also been evaluated for HIV prevention.[21] The intervention also used priming, which is when a stimulus, including sights, smells, and sounds, subconsciously or indirectly influences another behavior.[11, 22, 23] Priming occurs when simple associational cues activate aspects of one’s memory that then become more influential in processing new stimuli. For example, a fresh scent emitted at a medical center increased hand washing before patient exams from 51% to 80%.[24] However, despite a proliferation of studies using social norms and priming to change health and social behavior,[11, 19] these principles have never, to our knowledge, been used to improve the health of PLHIV.

We hypothesized that a low-cost intervention based on social norms and priming that was developed through an iterative, empathy-based design process could enhance TasP strategies by increasing clinic attendance and ART adherence. The objective of the pilot study was therefore to evaluate the feasibility, acceptability, and short-term effectiveness of the intervention on adherence and retention in care among adults attending two HIV clinics in Tanzania.

## Methods

### Study population

We conducted a quasi-experimental 6-month pilot study at two adult HIV care clinics in Shinyanga, a resource-constrained region in Tanzania where HIV prevalence is 7.4%.[25] “Clinic A” is a peri-urban government-run health center and “Clinic B” is a rural religiously-affiliated dispensary; approximately 1,200–1,500 PLHIV were receiving care at each clinic in the last quarter of 2015. The evaluation strategy included anonymous patient satisfaction surveys to determine intervention acceptability and feasibility and a quantitative pilot study to examine preliminary effects on retention in care and ART adherence after 6 months. Eligible participants were: 1) at least 18 years of age; 2) living with HIV infection; 3) and receiving HIV primary care at one of the two study clinics.

### Intervention development

The intervention development process used patient-centered design (“design-thinking”) and has been previously described.[26] Patient-centered design uses a creative, empathetic approach that draws on ethnographic methods and relies on rapid prototyping and user testing.[18] In brief, we used four approaches from design thinking: watching, listening, mapping, and testing. This process included disciplined clinic (n = 6) and household observations (n = 6), in-depth semi-structured (n = 19) and photo-based interviews (n = 5), a nominal group approach to brainstorming to avoid group-think,[27] and focus groups (n = 4) for feedback on iterative intervention designs. We identified five ‘patient segments,’ an idea derived from the concept of customer segments to guide business and marketing innovation, which describe a group’s perception of health, risk factors for poor adherence, and potential interventions. We created corresponding “journey maps” for each segment to represent stages in the treatment experience that influence adherence and potential areas for intervention.[26, 28]

We designed the intervention using insights derived from the formative research (Table 1). For example, we found that patients recognize one another at the clinic, offer support and advice, and typically ignore written materials (e.g., posters) encouraging adherence. We learned that fear of stigma causes some patients to go to great lengths to hide their treatment, such as placing ART in empty lotion or soap bottles. We observed sparse households with few non-essential objects except for outdated calendars, often used for decoration. In addition, we found that patients’ lives have no connection to the clinic between appointments. A monthly appointment is an anomaly, one day where everything revolves around living with HIV. For many this is a particular challenge as there is a high financial opportunity cost for taking a full day to visit the clinic and for some, a risk of disclosure. We determined that the ideal intervention would include a clinic component to leverage social norms and build social support, and a useful, inexpensive take-home component to increase engagement with health and the clinic between visits, and a recognizable, self-relevant prime that appeared on all intervention components.[29]

**Table 1:** Insights relevant to intervention development derived from the formative research phase, using tools from marketing research and patient-centered design.

Key Insights for Intervention Development
**1. Disclosure and stigma remain significant obstacles to antiretroviral therapy (ART) adherence and retention in care in Shinyanga, Tanzania.**
• People living with HIV infection will travel long distances to visit a clinic far from their community to avoid recognition. • Patients go to great lengths to hide ART during transport to and from the clinic. Although pills are distributed in bottles, many patients immediately disguise them. • Some patients ask strangers to pose as family members so that they can complete the first session of adherence counseling (which requires the presence of a treatment supporter) and subsequently initiate ART.
**2. Existing clinical programs and materials do not connect with patients’ personal motivations and beliefs for staying healthy.**
• The impact of living with HIV and taking ART on patients' social goals are inadequately addressed by providers (e.g., having children, getting married). • Patients do not look at materials in the clinic and typically cannot recall the content in these materials. In contrast, patients associate health and wellbeing with images from daily life including performing farm work (i.e., fetching water, harvesting crops) and sending children to school. Above all, people value images of strength and community—these images may motivate health behaviors. • Patients value traditional beliefs and approaches to maintaining health. • People have few belongings in their homes except for useful objects, and no patients retain materials from the clinic. Patients will keep some objects, such as calendars, after they are no longer useful if they value the look and feel of the item.
**3. Most of the time, patients living with HIV infection are disconnected from the clinic and relationships with providers and other patients.**
• Patients lack frequent touch points with the clinic and reminders to follow health recommendations. • The clinic is an informal source of social support as patients often encounter the same patients on their monthly visits. However, patients rarely interact with their peers outside of the clinic environment.
**4. Patients value a positive clinic environment, but clinics have few reliable norms in practice.**
• Patients value staff relationships and will travel out of their way to visit a clinic that has trustworthy and/or highly competent staff. • The behavior of staff is highly variable—some will congratulate patients, others scold. It is difficult to predict a visit experience. • Patients assume that many of their peers living with HIV are non-adherent.

### Intervention description

The final intervention included one clinic-based component to be implemented in both clinics and two take-home components. All components included the priming image of a Baobab tree (the “tree of life”), a positive image known by residents in Shinyanga where the trees are numerous. It is immediately recognizable, associated with health and medicinal properties, and regarded as a symbol of life and positivity in a landscape where little else can thrive. This image was paired with the saying “*Together we can hug the Baobab tree*”, a play on a widely known Tanzanian idiom about working together to achieve a goal. The saying was intended to convey the support available to patients in the clinic from each other, the staff, and the community.

The clinic-based component was an interactive poster that rewarded appointment attendance. Patients who attended three consecutive on-time visits were congratulated and given a colored sticker to place on a poster that was publicly displayed at the clinic. We developed four culturally relevant images, which we tested in focus groups, on which patients placed their sticker to collaboratively complete the picture; the accumulation of stickers conveyed that regular clinic attendance is normative. For example, one poster was in the shape of a Baobab tree and the stickers were the “leaves”. The prime image and motto was also on the poster. The stickers had Kiswahili words from which the patient could select, such as “brave” and “courageous”. In this activity, we expected the patient to feel positive emotions about his/her accomplishment and to experience pride at others seeing him/her place the sticker, while maintaining anonymity.

We developed two take-home components, one for each clinic. In Clinic A the take-home component was a 2015 calendar in Kiswahili that contained the priming Baobab image and idiom. The calendar contained six images, inspired by the photography exercise, that were confirmed in focus groups to be associated with health, strength, and well-being, such as carrying water, children in school uniforms, farming, and infants. The calendar had the potential secondary benefit of helping people keep track of their appointments. In Clinic B the take-home component was a small plastic pillbox featuring the Baobab logo and idiom. We hypothesized that a simple pillbox (<$0.50) may alleviate some of the stress associated with inadvertent HIV disclosure, therefore improving adherence. We selected a 4-compartment case (1 3/8" x 2 3/4" x 5/8") that could hold several ART doses (depending on regimen). The pill case could fit in a pocket and was intentionally shaped like a feature phone, another measure to prevent inadvertent disclosure of HIV status.

### Sampling and data collection

We collected data from two samples of adult patients. First, patient satisfaction was measured with an anonymous survey at baseline and endline (6 months). At both time points, the research team approached a convenience sample of patients at the adult (≥18 years) HIV clinic who were asked to complete a brief in-person interview about their clinic experience (baseline and endline) and perceptions of the intervention (endline only).

Second, we conducted a quasi-experimental, 6-month pilot study at the two HIV treatment clinics. Because the intervention was delivered at the clinic level, we could not randomly assign individuals to the intervention. Instead, we assigned groups of individuals to the intervention by implementing the intervention during two weeks of each month, with the other two weeks per month as the comparison (standard of care) weeks. Because most PLHIV on ART in Tanzania follow strict 30- or 60-day visit schedules, this design assured that most (but not all) participants were exposed to only the intervention or the standard of care when they returned to the clinic for their regularly scheduled visits. The intervention began August 10^th^, 2015 and was implemented every two weeks for 6 months.

This was a pilot study to primarily determine intervention acceptability and feasibility, therefore we did not conduct formal sample size calculations. We determined that it was feasible to compare outcomes among approximately 400 patients, with the assumption that data would be available for approximately 80% of patients. Patients were sampled using systematic random sampling with a random start and a sampling interval in order to yield a final sample that oversampled exposed patients. The final number sampled in each group depended on the number of patients in each sampling frame at each clinic. We first used intervention logs that we maintained to sample 393 patients who were exposed to the intervention in the first two intervention periods, representing a mix of patients who received both a sticker and a take-home component at baseline (100% of this “fully exposed” group was sampled, n = 193) and those patients receiving only a take-home component at baseline (100 sampled per clinic) to ensure adequate representation in the sample. In the analysis, we combined these groups because patients who were partially exposed at baseline (by virtue of only receiving a take-home component) often moved into the “fully exposed” group during the intervention period, so a single, combined intervention group was deemed most appropriate. We then used the pharmacy dispensing register to select a sample of 149 unexposed patients who attended a clinic visit in the first standard of care period. For all sampled patients with available records, we abstracted visit attendance and pharmacy dispensing data from medical registers for the six months following the sampled visit and the two appointments before the intervention period.

### Outcomes

Intervention feasibility and acceptability was determined by questions directed to patients about staff support for treatment and life goals, overall satisfaction with care received at the clinic, and their experience with intervention components. The primary outcomes in the pilot study were retention in care 6 months after baseline, defined as a visit between 150 and 210 days after the baseline visit (6 months +/- 30 days), and ≥95% adherence to ART, measured with the medication possession ratio (MPR). MPR is the proportion of days in an interval that an individual was in possession of ≥1 dose of ART, according to pharmacy dispensing data and is highly correlated with viral suppression.[30] When patients transferred or died, we truncated the 0–6 month interval to the last visit date. Otherwise the denominator of MPR included all days in the 0–6 month interval. Secondary outcomes were “appointment adherence,” the proportion of scheduled visits that were completed during the 6-month observation period,[31] and MPR on a continuous scale.

### Data analysis

We first describe responses to questions on the anonymous patient satisfaction survey and compare baseline and endline values with Pearson’s chi-square test. We then examined whether there were baseline differences between the intervention and comparison groups in the pilot study by applying weights to account for the stratified sampling strategy and using t-tests to compare means (i.e., MPR, appointment attendance) or survey design-corrected Pearson’s chi-square test to compare proportions.[32] We constructed unadjusted logistic (i.e., MPR≥95%, in care at 6 months) and linear (i.e., MPR, appointment attendance) regression models to determine the association between intervention exposure, as defined by week of clinic attendance at baseline, and the primary and secondary outcomes and report odds ratios (ORs) and mean differences, respectively, with 95% confidence intervals (CIs). We repeated this analysis after adjusting for clinic, which was imbalanced between study groups. These analyses excluded 104 of 542 sampled patients: patients <18 years (n = 24), patients not yet on ART (n = 6), patients without medical records (n = 73), and patients (n = 1) who transferred before the first scheduled visit. The proportion of excluded patients did not differ by study group (21% comparison vs. 20% intervention, p = 0.66). All analyses were weighted to account for the sampling strategy and conducted with STATA v.14 (College Station, Texas).

We also conducted a sensitivity analysis to explore the effect of variable appointment scheduling and unscheduled visits on exposure to the intervention and its effectiveness. Specifically, we found that during the 6 month intervention period, 71% and 21% of individuals in the intervention and comparison groups, respectively, were exposed to the intervention during at least half of their attended visits. To account for this imperfect treatment exposure and the potential for confounding factors affecting both outcomes and actual treatment exposure, we used an instrumental variable (IV) approach to estimate the intervention’s effectiveness on the primary outcomes.[33] In these 2-stage least squares regression models, treatment assignment (as determined by baseline visit week) was an instrument for the actual treatment exposure, defined as ≥50% of attended visits occurring during intervention periods.

### Protection of human subjects

The Tanzanian National Institute of Medical Research and the Committee for Protection of Human Subjects at the University of California, Berkeley approved this study. Written informed consent was obtained from participants in in-depth interviews and focus group discussions conducted as part of the formative research. Verbal informed consent was obtained from participants completing patient satisfaction surveys. Because the intervention was delivered at the clinic level to all patients, was intended to be a subtle cue and display of information, involved minimal to no risk, and could be evaluated using de-identified existing data, both ethical review boards determined that informed consent of participants in the pilot study would be impractical and therefore granted a waiver of informed consent. The quasi-experimental pilot study was registered in Clinicaltrials.gov (NCT02938533, https://clinicaltrials.gov/show/NCT02938533) after retrospective data collection of existing data was complete in order to conform with the International Committee of Medical Journal Editors policy. The authors confirm that all ongoing and related trials for this intervention are registered.

## Results

### Intervention monitoring and implementation

During the six-month intervention period (August 10^th^–January 22, 2016), we implemented three interactive posters at each clinic, each displaying a different image which participants “filled” with stickers. We distributed 500 calendars (Clinic A) and 500 pillboxes (Clinic B) during the first two intervention periods to clinic patients.

### Intervention acceptability and feasibility

Overall, 405 patients completed the anonymous patient satisfaction survey (189 at baseline and 216 at endline). The median age was 40 years, 61% were female, and 51% attended clinic A; there were no significant differences between participant characteristics at baseline and endline. Compared to baseline (Table 2), at endline we found significant increases in staff support of treatment goals (100% vs. 95%) and life goals (66% vs. 50%), the perceived likelihood of other patients’ high levels of adherence (54% vs. 32%), support from other patients (71% vs. 60%) and being very satisfied with clinical services and care (53% vs. 35%). There were significant decreases in unanswered questions for providers (25% vs. 38%). At endline, patients who reported ever viewing the poster more often reported that they would “definitely” take all of their ART the following month (99%) compared to patients who had not viewed the poster (93%, p = 0.03). Furthermore, the proportion of patients who were “very satisfied” with care was non-significantly higher among patients who reported viewing the poster (56%) compared to those who had not viewed the poster (44%, p = 0.11). Open-ended questions revealed that patients found the interactive poster and process of earning a sticker acceptable, noting that it made them feel “good for being recognized” as a “hero” or “champion.” Among pillbox recipients, all (100%) reported using the box to store treatment. Among those who received a calendar, 85% reported using it to track appointments.

**Table 2:** Patient satisfaction at baseline and after the six-month intervention period (endline), Shinyanga, Tanzania, 2015–2016.

Characteristic		Adult Patients at Baseline (n = 189)	Adult Patients at Endline (n = 216)	χ^2^	p-value
		n (%)	n (%)		
**Clinic Environment**						
**Satisfaction with services and care**						
** **	Less than satisfied	7 (3.7)	5 (2.3)	14.2	<0.01	
** **	Satisfied	116 (61.7)	96 (44.4)			
** **	Very satisfied	65 (34.6)	115 (53.2)			
**Enjoy being at the clinic**						
** **	Disagree/Strongly disagree	35 (18.5)	29 (13.5)	1.9	0.17	
** **	Agree/Strongly agree	154 (81.5)	186 (86.5)			
**Other patients support me**						
** **	Disagree/Strongly disagree	75 (39.7)	63 (29.2)	5	0.03	
** **	Agree/Strongly agree	114 (60.3)	153 (70.8)			
**Patient-Provider Interactions**						
**Staff support treatment goals**						
** **	Not supportive	9 (4.8)	0 (0)	10.7	<0.01	
** **	Supportive	99 (52.4)	123 (56.9)			
** **	Very supportive	81 (42.9)	93 (43.1)			
**Staff support life goals**						
** **	Not supportive	95 (50.3)	73 (33.8)	12.8	<0.01	
** **	Supportive	81 (42.9)	114 (52.8)			
** **	Very supportive	13 (6.9)	29 (13.4)			
**Unanswered questions today**						
** **	Yes	72 (38.1)	53 (24.5)	8.7	<0.01	
** **	No	117 (61.9)	163 (75.5)			
**Given instruction by pharmacist today**						
** **	Yes	95 (50.3)	134 (62.0)	5.7	0.02	
** **	No	94 (49.7)	82 (38.0)			
**Understanding of instructions**						
** **	Understood some/none	4 (4.2)	5 (3.8)	0.03	0.86	
** **	Understood all	91 (95.8)	128 (96.2)			
**Patient Adherence**						
**Likelihood of attending scheduled visit**						
** **	Definitely	175 (92.6)	200 (92.6)	0	1	
** **	Not sure/Probably not	14 (7.4)	16 (7.4)			
**Likelihood of taking all ART this month**						
** **	Definitely	180 (95.2)	210 (97.2)	1.1	0.29	
** **	Not sure	9 (4.8)	6 (2.8)			
**Likelihood of others taking all ART**						
** **	Most	60 (31.7)	116 (53.7)	25.8	<0.01	
** **	Some	36 (19.1)	36 (16.7)			
** **	Few	18 (9.5)	22 (10.2)			
** **	Not sure	75 (39.7)	42 (19.4)			

### Retention in care and ART adherence

**Fig 1:**
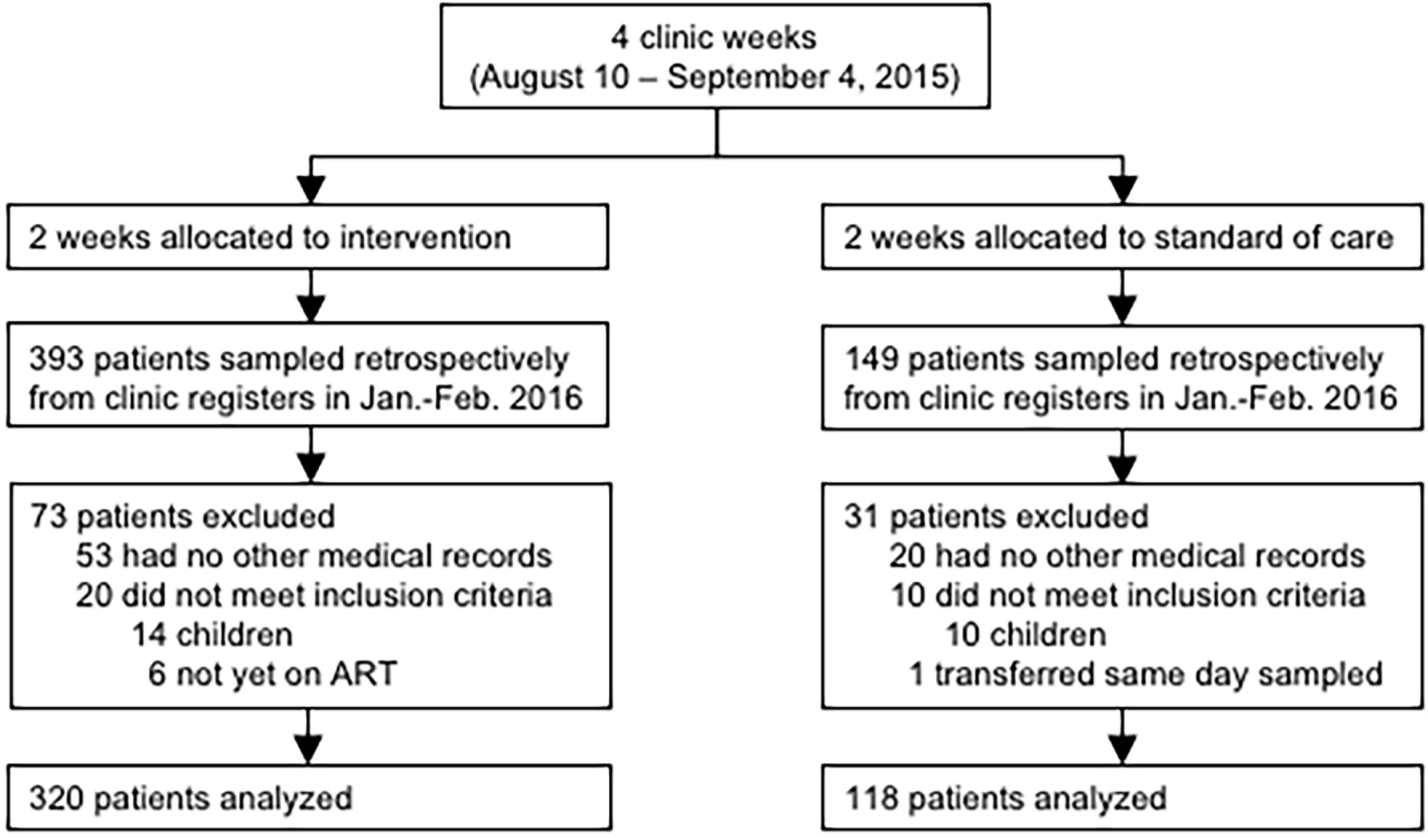
Study flowchart.

Medical records were reviewed for 438 randomly sampled patients, 320 exposed to the intervention and 118 receiving standard of care, from January 27, 2016 to April 26, 2016 (Fig 1). At baseline, age, sex, time on ART, regimen and appointment adherence were similar in the intervention and comparison groups, whereas Clinic B was overrepresented in the treatment group (62% vs. 55%, p<0.01, Table 3). During the study period, MPR≥95% was achieved by 64% of sampled patients, 83% of patients remained in care after 6 months, and mean appointment attendance was 69%.

**Table 3:** Characteristics of adult HIV primary care clinic patients, stratified by intervention exposure at baseline, Tanzania, 2015–2016.

Characteristic^a^		Intervention	Comparison	Test statistic^e^	p-value
		N = 320	N = 118		
**Age (%)**					
** **	18–29	0.19	0.16	0.27	0.84
** **	30–39	0.32	0.35		
** **	40–49	0.28	0.27		
	≥50	0.21	0.22		
**Sex (%)**					
** **	Female	0.62	0.55	2.93	0.09
** **	Male	0.38	0.45		
**Time on ART (%)**					
	≤90 days	0.13	0.10	2.41	0.07
	3–12 months	0.19	0.14		
** **	1–3 years	0.28	0.39		
** **	>3 years	0.40	0.37		
**ART initiate (%)**^b^					
** **	Yes	0.13	0.10	0.98	0.32
** **	No	0.87	0.90		
**Baseline Appointment Attendance (%)**^c^		0.74	0.73	0.09	0.93
**Regimen (%)**^d^					
	3TC(150mg)/AZT(300mg)/NVP(200mg)	0.11	0.08	0.83	0.47
	AZT(300mg)/3TC(150mg)/EFV(600mg)	0.12	0.12		
** **	TDF(300)/FTC(200)/EFV(600mg)	0.09	0.13		
** **	TDF(300)/3TC(300)/EFV(600mg)	0.68	0.67		
**Clinic (%)**					
** **	Clinic A	0.38	0.45	12.29	0.001
	Clinic B	0.62	0.55		

a. Weighted statistics to account for sampling strategy. Missing data for weighted patient sample: 2% age, 2% sex, 3% time on ART, 1% baseline appointment attendance, and 3% regimen.

b. Started ART ≤90 days before baseline.

c. Attendance at the last two scheduled appointments (+/- 4 days) prior to the baseline visit.

d. Regimen recorded at the baseline visit.

e. Survey design-corrected Pearson’s Chi-square test for categorical variables and t-test for continuous variables.

In unadjusted and adjusted analyses, PLHIV exposed to the intervention were significantly more likely to be in care after 6 months (87% vs. 79%, OR_a_ = 1.73, 95% CI: 1.08, 2.78, p<0.05, Table 4) and were more likely to achieve MPR≥95% (70% vs. 59%, OR_a_ = 1.51, 95% CI: 0.96, 2.37, p = 0.07), although this result was not statistically significant after adjustment for clinic. The intervention and comparison groups had similar average MPRs (90% vs. 89%, adjusted mean difference = 0.0, 95% CI: -0.04, 0.04, p = 1.0) and appointment adherence (73% vs. 66%, adjusted mean difference = 0.04, 95% CI: -0.01, 0.09, p = 0.09). When stratified by clinic, the effects were concentrated at Clinic A, where we observed a 16 percentage point increase in retention in care at 6 months (83% vs. 68%, OR = 2.38, 95% CI: 1.28, 4.44, p<0.01), a 24% increase in retention. The sensitivity analysis using an IV analysis approach generated similar results to the main analysis; results are presented as Supporting Information (S1 Table).

**Table 4:** Crude and adjusted associations between intervention exposure and retention in HIV care and adherence to antiretroviral therapy after 6 months, Tanzania, 2015–2016.

	Mean (95% CI) at 6 months ^a^		Estimated Associations			
	Intervention (N = 320)	Comparison (N = 118)	Unadjusted		Site-Adjusted^g^	
			OR^e^	(95% CI)	OR_a_	(95% CI)
**Retained in care**^b^	0.87 (0.85, 0.90)	0.79 (0.73, 0.86)	1.83	(1.16, 2.91)**	1.73	(1.08, 2.78)*
**MPR ≥95%**^c^	0.70 (0.65, 0.74)	0.59 (0.50, 0.67)	1.62	(1.08, 2.41)*	1.51	(0.96, 2.37)
			Mean Diff^f^ (95% CI)		Mean Diff_a_ (95% CI)	
**MPR**^c^	0.90 (0.87, 0.92)	0.89 (0.86, 0.92)	0.01	(-0.03, 0.05)	0	(-0.04, 0.04)
**Visit Attendance**^**d**^	0.73 (0.70, 0.75)	0.66 (0.61, 0.71)	0.06	(0.01, 0.12)*	0.04	(-0.01, 0.09)

CI: confidence interval, OR = Odds ratio

*p≤0.05

**p≤0.01

a. Weighted analyses to account for sampling strategy.

b. Whether the patient was in care at 6 months, defined as a visit between 150 and 210 days (6 months +/- 30 days). Excluding <1% of weighted sample who transferred before 6 months.

c. Medication possession ratio, the proportion of time an individual is in possession of ≥1 ART dose. Excluding 21% of weighted sample who were missing dispensing data for at least one recorded visit.

d. The proportion of scheduled appointments attended (+/- 4 days) during the 6-month intervention period. Excluding <1% of weighted sample who were missing all scheduled visit records.

e. Based on logistic regression by treatment.

f. Based on linear regression by treatment.

g. Adjusted for clinic site.

## Discussion

We developed and conducted a pilot evaluation of a combined intervention inspired by the concepts of social norms and priming to improve retention in care and adherence to ART among people living with HIV infection in Tanzania. We found that the intervention was feasible and acceptable to patients and was associated with temporal improvements in the perceived level of support from staff and other patients as well as improvements in satisfaction with clinic services. Results from the quasi-experimental pilot study indicate that the intervention was associated with better retention in care and potentially a higher proportion of PLHIV achieving MPR≥95% over a short 6-month follow-up period. These promising findings suggest that the application of innovative, low-cost approaches from behavioral economics and psychology, such as social norms and priming, may enhance the HIV care continuum and bolster the effectiveness of ART programs, including TasP.

The combination intervention included components that were intended to leverage multiple motivational pathways to influence a diverse group of PLHIV. First, the intervention leveraged social norms through an interactive poster intended to motivate PLHIV to attend clinic appointments and pick up ART from the pharmacy. Several studies have demonstrated the power of social influence,[11, 19] which is the idea that individuals use norms to compare and adjust their own behavior.[34] The interactive poster’s creative visual feedback about norms was deisgned to allow patients to benchmark their adherence behavior against others attending the same clinic, and potentially incite better visit attendance and/or adherence. Although we do not know the exact mechanism of action, after our intervention, patients were significantly more likely to say that *other patients* would take most of their ART in the next month, a finding that may be linked to the interactive poster which demonstrated that adherence to scheduled appointments is normative. The poster may have also improved the overall clinic experience, by recognizing the accomplishments of PLHIV, eliciting pride and social support when one earned a sticker and physically placed it on the poster in view of fellow patients. In addition, this investment in the social fabric of clinics may have created a motivating and positive clinic environment. These hypotheses may warrant further investigation.

The intervention was also designed to include a prime that was *identity-relevant*, meaning that the cue was related to one’s self-concept (e.g., gender, race, or group membership, such as PLHIV) and therefore hypothesized to stimulate ‘identity congruent’ behavior.[35] For example, people buy products or behave in a manner that is consistent with their self-image, despite not being consciously aware of the stimuli’s influence. We hypothesize that the Baobab logo and idiom about social support was relevant to patients’ identity as a person living with HIV infection in Shinyanga, and reminded them that they have a support system, even if they had not disclosed their HIV-infected status to friends and family. PLHIV in the study had many opportunities for exposure to the prime: the image appeared on posters, pillboxes, and calendars, and Baobab trees are ubiquitous in Shinyanga. This may have subconsciously motivated PLHIV to attend visits and retrieve ART before their supply was exhausted. Only 13% of the patients surveyed could recall the Baobab logo, suggesting that the prime, if was effective, operated primarily outside of conscious awareness as intended.[22] However, neither our intervention nor experimental approach was singularly designed around assessing the impact of the prime alone, and instead treats the image as one component of a combination intervention.

Lastly, our empathetic approach to intervention design led to practical take home components (calendars and pillboxes) that built upon people’s natural behavior and preferences and subtly displayed the priming image. Formative research indicated that patients were already hiding their pills; the pillbox intervention made this activity simpler. Similarly, patients already displayed old calendars in their home; the new ones simply stimulated health behaviors in a subtle way through images. Both calendars and pillboxes are considered “passive reminder devices” that can improve adherence to ART.[36] Together, the low-cost intervention components were intended to subtly motivate a heterogeneous group of PLHIV to remain engaged in HIV care.

The intervention’s effects were strongest at a busy, government-run clinic (Clinic A) where patients received a calendar as the take-home component. At this clinic, our formative research revealed that patients travel very far and often couple their clinic visit with a visit to large nearby market, which provides a useful (non-HIV related) explanation for being away from home. Furthermore, we found that patient-provider interactions at Clinic A were often negative–which suggested that a simple intervention to improve the clinic experience and remind patients about attendance may work well. In contrast, at Clinic B where the effects were weaker, patients benefitted from staff who were inherently more service oriented, even introducing a new lunch program during the study whereby patients attending three consecutive scheduled visits were provided with a coupon for a free lunch at a local establishment. Although we were pleased that the clinic appreciated and duplicated the strategy to recognize clinic attendance–perhaps a reflection of the inclusive design approach–this competing program offered to all patients may have contaminated the standard of care group, thereby potentially explaining the lack of a strong effect at Clinic B. Nevertheless, future studies are needed to understand the influence of the clinical setting and participant characteristics on the success of approaches using behavioral economics and/or psychology. It may also to be important to understand the influence of the specific take-home component (i.e., pillbox, calendar) on these results, which was beyond the scope or intention of this pilot study but could inform the design of future interventions.

This study has several limitations. First, the intervention was designed and evaluated among patients attending two clinics in Tanzania. Future studies will need to understand the generalizability of the approach and rigorously measure its effectiveness on clinical outcomes. In addition, given provider variability in scheduling appointments and unscheduled visits, some patients in the intervention group attended clinic appointments during non-intervention weeks, and vice versa. Although this resulted in some contamination of the comparison group, results from our sensitivity analysis using an instrumental variable approach support our primary finding that the intervention improved retention in care and potentially ART adherence (see S1 Table). Spillover effects are also possible, albeit unlikely, if intervention group clients told peers about the intervention and that information changed the adherence behavior of comparison group clients. Furthermore, we are unable to make statements about the effectiveness of individual intervention components given that the intervention was designed as a combination package. Future studies could consider whether individual components warrant independent evaluation. We did not measure viral suppression, the ultimate goal of HIV treatment. However, MPR is closely correlated with short-term viral load.[30, 37] In addition, as with many studies in resource-constrained settings, there is the potential for poor data quality and missing data in paper-based facility registers; however, missing data was not differential by study group. Furthermore, missing data would result in our study *underestimating* ART adherence and retention in care. Lastly, patients self-reported satisfaction and how they used the take-home components, which might be affected by social desirability bias.

Our study also has significant strengths, including being the first of its kind to apply the principles of social norms and priming to ART adherence and retention in care. We used tools from marketing research and patient-centered design[18] to create an intervention that is responsive to the needs of PLHIV, and we evaluated its acceptability and potential effectiveness in a quasi-experimental pilot study. The evaluation suggests that the intervention has the potential to improve retention in care and adherence to ART over 6 months. These findings are important not only for individual patients, due to the increased likelihood of HIV care and viral suppression, but also for the health system due to increased value for money of the treatment program. Governments and donors have invested millions of dollars into TasP programs; with a minimal budget, we increased retention and adherence, which could translate to many more years of life given the scale of TasP programs. Future evaluation of this design and intervention approach is warranted, especially in different geographic areas and with various health outcomes.

## Supporting information

S1 Data Medical record data: Metadata. (CSV)

S2 Data De-identified medical record data. (CSV)

S3 Data Patient surveys: Metadata. (CSV)

S4 Data De-identified patient survey data. (CSV)

S1 File Study protocol. (PDF)

S2 File Study survey guides. (PDF)

S3 File TREND statement. (PDF)

S1 Table Results of sensitivity analyses to determine intervention effectiveness accounting for imperfect treatment exposure. (DOCX)
