# Supplementary material for: Pilot study of a multi-pronged intervention using social norms and priming to improve adherence to antiretroviral therapy and retention in care among adults living with HIV in Tanzania
Source: PLoS One. 2017 May 9;12(5):e0177394. doi: 10.1371/journal.pone.0177394 (PMC5423659; doi:10.1371/journal.pone.0177394)
Supplement: S1 File — (PDF) [file pone.0177394.s005.pdf]

STUDY PROTOCOL

Pilot Study of a Multi-Pronged Intervention Using Social Norms and Priming to Improve Adherence to Antiretroviral Therapy and Retention in Care Among Adults Living with HIV in Tanzania

STUDY TEAM

Principal Investigator

Dr. Sandra McCoy
Assistant Adjunct Professor, Division of Epidemiology
School of Public Health
University of California, Berkeley
779 University Hall, MS 7360
Berkeley, California, 94704
Phone: +1 (510) 642-0513
Email: smccoy@berkeley.edu

Co-Investigators

Dr. Ntuli Kapologwe
Regional Medical Officer, Shinyanga Region
Ministry of Health and Social Welfare (MOHSW) Tanzania
Shinyanga Regional Hospital
P.O. Box 17
Shinyanga, Tanzania
Phone: +255 (784) 597-929, +255 (713) 855-098
Email: Kapologwe@gmail.com

Dr. Prosper Njau
Program Officer for Prevention of Mother to Child Transmission of HIV (PMTCT)
Ministry of Health and Social Welfare (MOHSW) Tanzania
Directorate of Preventive Services, Reproductive and Child Health Section
MOHSW Headquarters, Junction Samora Avenue and Luthuli Street
Postal Box 9083
Dar es Salaam, Tanzania
Phone; +255 (784) 597-929, +255 (713) 855-098
Email: prosperpendo@gmail.com

Mr. Sergio Bautista-Arredondo
Health Economics Department
National Institute of Public Health
AV UNIVERSIDAD 655
Cuernavaca, Mexico
Phone: (777) 329-30-68
Email: sbautista@insp.mx

SUMMARY

This project is an interdisciplinary two-phase study that will evaluate an innovative behavioral intervention to encourage HIV-infected patients in two clinics in Shinyanga Region to adhere to antiretroviral therapy (ART). In the first phase of the study, researchers will draw on patient-centered

design and marketing research tools to identify important motivations, influences, and aspirations of the target population in order to design a low cost intervention based on the concepts of priming and social norms. In the second phase, researchers will implement the intervention in two clinics and gather data to see whether there is preliminary evidence that the intervention is successful in helping patients adhere to ART and remain in HIV care. If so, this study will lay the groundwork for a more comprehensive impact evaluation of the approach.

INTRODUCTION AND MOTIVATION

HIV prevalence in Tanzania is 5.1%, and although HIV care and treatment services are widely available to the 1,400,000 people living with HIV infection, poor adherence to ART is a pervasive threat to its effectiveness.[1] Indeed, nearly one-quarter of patients in sub-Saharan Africa fail to achieve adequate levels of adherence,[2] a challenge that undermines both the clinical benefits of ART and its public health value of reduced secondary transmission.[3] Although several strategies have been identified to improve adherence among PLHIV in sub-Saharan Africa, most are time, labor, or financially intensive, including directly observed therapy, mobile phone text messages, cash incentives, and food rations.[4] Such interventions have unclear prospects as scalable interventions that can seamlessly integrate within existing health systems. However, based on theories from psychology and behavioral economics, we hypothesize that a simple, innovative, and inexpensive strategy using behavioral priming and social norms may be equally effective at improving ART adherence and more cost-effective than other approaches.

Behavioral *priming* occurs when a stimulus subconsciously or indirectly influences another behavior[5], and can include sharing information in a new way or displaying subtle associational cues. The concept of behavioral priming has its origins in a landmark social psychology study, which revealed that activating a trait, construct, or belief, for example by exposing subjects to words related to old age and slowness, elicits behavioral effects in study subjects such as walking more slowly.[6] Psychological theory suggests that behavioral effects are most likely when an individual already has a situational association with the priming stimulus—for example, a logo for a soda company is more likely to elicit a behavioral response from people familiar with that logo.[7]

The power of priming is best illustrated by the compelling results from experimental studies. People can be primed using simple associational cues whose effects may not necessarily be intuitive; for example, people are more creative after seeing the Apple versus Microsoft logo and, when there's a faint scent of cleaning products in the air, people tidy up better after eating a crumbly cookie.[8] Subtle changes in the presentation of data can also prime people to act differently: a study in Kenya found that an abstinence-only curriculum for teenagers, including general HIV prevention information, was ineffective, but a simple one-time session where the percent of men with HIV infection in different age groups was written on the blackboard resulted in a 28% decrease in teen pregnancy, a marker for unprotected sex.[9] More recent observational studies suggest that changing patients' environments to mimic the circumstances of their youth can help revitalize health.[10] Although these studies suggest the potential for subtle, carefully crafted stimuli and messages to motivate people to engage in and maintain desired behaviors, these principles have never been used to improve adherence to ART.

The intervention will also draw on concepts related to social norms and social influence, which are increasingly recognized as powerful behavior change tools.[1112] For example, a recent study reduced inappropriate antibiotic prescribing by informing frequent prescribers that they were prescribing at a higher rate than 80% of other practices.[13] After the intervention, there was a 3.3% reduction in antibiotic prescribing, translating to 73,406 fewer prescriptions during the short 6-month study period.

Furthermore, energy bills printed with a simple comparison of a household's energy use to their neighbors along with encouraging smiley faces are highly effective at cutting energy consumption, even more so than high-cost investments like smart meters.[14] In the health domain, informing college students who drink heavily about the lower average consumption of their peers is an effective strategy to curtail binge drinking.[15]

In the context of adherence to ART, interventions using behavioral priming and social norms might include redesigned pill packaging, posters, communications, or data visualizations summarizing the clinic's overall adherence rate. These low-risk interventions do not change a patient's treatment or treatment plan, but instead may help to subconsciously motivate them to remain adherent. This type of behavioral approach is well suited to HIV care and treatment clinics in sub-Saharan Africa, where patients often wait for long periods of time and sometimes receive stigmatizing treatment by healthcare providers. Consequently, there is ample opportunity to replace the negative associations from this experience with positive ones promoting adherence.

In this research study, the intervention(s) will be developed in **Phase 1** and evaluated in a pilot study in **Phase 2.** The two sequential phases are described below:

Phase 1: Phase 1 will focus on designing the priming intervention(s). In the two selected study sites, we will use a combination of qualitative and ethnographic data collection approaches as well as tools used by marketing research to design the intervention. This will include an independent photo activity followed by 5 in-depth semi-structured interviews based on photos collected, general in-depth semi-structured interviews with 25 HIV clinic patients, and household observations of 10 patients. These formative research activities are described in greater detail in the Methodology section below. Based on these data, the team will develop one or more simple priming interventions that will be tested and refined in up to 5 focus group discussions (FGDs).

Phase 2: In Phase 2 we will conduct a 6-month pilot study at the same two HIV care and treatment clinics. At each clinic, we will implement the intervention during the first two weeks of each month for 6 months, with the last two weeks per month as the comparison (standard of care) weeks. To determine the potential effectiveness of the intervention, we will use a parallel group prospective cohort design. We will randomly sample PLHIV from each clinic register who had visits during the intervention weeks and comparison weeks and we will measure whether they remained in care and had improved adherence to ART after 6 months. We will compare these outcomes between the two groups: those exposed to the intervention and those unexposed to the intervention.

At the conclusion of the study, we will have evidence about whether this is an acceptable, feasible, and potentially effective approach to improve adherence to ART in Sub-Saharan Africa.

OBJECTIVES

The following are the primary objectives of this study:

1) Develop a low cost and effective intervention using behavioral priming and social norms to help patients better adhere to ART and remain in HIV care.
2) Evaluate the preliminary effectiveness of the intervention in a 6-month pilot study.

METHODOLOGY

The goal of this study is to develop a behavioral priming intervention(s) that encourages PLHIV in Shinyanga, Tanzania to adhere to their treatment drug regimens. Because data collection is divided into a formative research phase and pilot impact study, the chronology will be laid out along these dimensions. The formative research (Phase 1) precedes the pilot study (Phase 2).

Phase 1: The formative research phase will employ three qualitative data collection methods: 1) photo collection and photo-based interviews 2) 25 interviews plus home observations for a subset (n=10) of interviewees; and 3) five focus group discussions with 6-8 people each. The purpose of these qualitative research activities is to learn about what factors motivate good health and wellbeing for HIV positive patients visiting care and treatment clinics, identify elements of their daily lives that influence their care and treatment, understand the barriers for patients successfully adhering to their treatment over time, and uncover subtle aspirations, influences, and personal motivations that could be leveraged for a priming intervention. Because priming and social norm interventions can potentially draw on a broad set of aspirations, influences, and motivations to influence behavior, many interview questions will not be directly related to health. Taking a broad based approach to understanding the underlying factors for an individual to use or not use a product/service is a common marketing approach for firms designing successful advertising campaigns, product packaging, and other collateral. We will adapt this approach to create patient journey maps and fictional personas that showcase similar information for a person's likelihood to adhere to ART. By examining the experience of patients receiving ART and maintaining treatment over time, we can design more successful interventions that tap into aspirations and motivations that may or may not be directly related to health.

Recruitment: For both interviews and focus group discussions, research staff will review the daily appointment log at the care and treatment clinics and will alert the clinic staff of potentially eligible participants who are ≥18 years of age. We will aim to balance gender, duration of ART treatment, and adherence practices in our selection of potentially eligible participants. When potentially eligible patients check-in for their appointment, they will be asked by clinic staff whether they would like to hear more about a research study. If yes, the clinic staff member will alert a member of our Swahili-speaking research team who will approach patients using a recruitment script. If a patient is willing to hear more about the study, we will begin the process of informed consent. In brief, the research assistant will explain the procedures, will explain that participation is voluntary, that their participation and responses will not alter the care they receive at the clinic, and that we will take all efforts to protect their rights and confidentiality.

In this first phase of the study we will explain to subjects that the study aims to strengthen HIV/AIDS treatment services in the region and understand the factors that motivate health and wellbeing rather than discussing the concept of behavioral priming and social norms in detail. This is for two reasons: 1) priming and norms are complicated topics to explain clearly, and a thorough understanding is not necessary to safely participate in the study, and 2) because priming is about sub-conscious behaviors, explaining it fully may bias subjects' responses to interview and FGD questions and alter their behavior during observations.

Our specific methodology for each approach is as follows:

1. <u>Five In-depth interviews based on photo collection:</u> After we have obtained written informed consent, we will provide the subject with a disposable camera that includes 25-30 exposures. The subject will have five days to take pictures of anything that reminds him/her of positive emotions such as happiness, health, and wellbeing. Given this directive, we don't expect the photos to reveal anything identifiable about the participant's HIV status, contact information, or other protected

health information. We will explain that subjects should not photograph other people unless they have received verbal permission. After five days, we will collect the cameras and develop the pictures at a local photo shop. We will only label the cameras with numbers so that they do not include identifiable information. We will schedule an in-depth interview that will take place at a private space at the clinic (or nearby in a quiet space outdoors) to share and discuss the photographs. The interviews will last approximately 1 hour and follow a semi-structured interview guide. Interviews will be audio recorded with the participant's consent.

<u>2. 25 In-depth interviews and 10 home observations:</u> After we have obtained written informed consent, the in-depth interview can be conducted on the same day as the appointment in a private space in the clinic or outdoors. The interviews will last between forty-five minutes to an hour and a half. The questions will draw from a semi-structured interview guide, but the interviewer may probe more deeply on a specific topic that arises in conversation, consistent with the principals of qualitative data collection.[16] Interviews will be audio recorded with the participant's consent.

During the informed consent process, the interviewer will ask the participant if s/he is also willing to participate in a household observation, in addition to the interview. If the patient agrees then the interviewer will arrange a time for the visit and note down the patient's contact information. The household observations will last between four to six hours and consist of mostly passive observation. With the participant's consent the researcher will visit the participant's home in the morning to observe how his/her day begins. This type of observation is a common technique used in business and marketing to understand how people interact and think about a particular product or service, in this case, antiretroviral therapy. We will use a note-taking framework to take notes on the participant's day.

The researcher will not influence what activities the subject does and will only ask clarifying questions about why a subject does a particular activity, how often s/he does it, and what s/he is thinking as s/he engages in the activity. Although the researcher will encourage the subject to continue his/her daily routine without making exceptions for the researcher, we recognize that the mere presence of an unfamiliar person may cause the subject to act differently. The researcher will do his/her best to minimize this by occupying an inconspicuous space, but it will be impossible to entirely mitigate this effect. Despite this, we believe that we can gather useful information from the observations such as where the subject stores his/her ART, what events might be taking place while subjects try to take medications, daily schedules, etc.

No audio or video footage will be recorded from the observations. The researcher will only enter areas of the subject's home and land where the subject has granted permission. The goal is to create a map of how, when, and why people think about their health. We will conduct up to 10 home visits.

<u>3. Five FGDs:</u> We will conduct FGDs once we have developed preliminary priming interventions based on data collected from the in-depth interview and home visits. We will use the recruitment strategy described above and will follow the same procedures to obtain written informed consent. After we have obtained written informed consent, the FGDs will be scheduled for the same day or a future day when 6-8 participants are available. In the FGDs, participants will be provided with product or intervention samples (these might include new pill packaging, posters, etc.) and researchers will gauge their reaction to the proposed intervention. Research staff will explain that any information shared within the group is confidential, but no sensitive questions will be asked (see sample guide, attached). The FGD will be closer to a product demonstration used in business and

marketing than a classical FGD. Nevertheless, participants may spontaneously divulge information about themselves or their community, so we will follow strict procedures for ensuring confidentiality. Discussions will last 1-2 hours in length and will be audio recorded with participants' consent.

Phase 2: In Phase 2, we will implement and evaluate the effect of the intervention(s) that will be developed in Phase 1.

A. The intervention: Our formative research led us to develop the following intervention based on the concepts of priming and social norms that will be tested in Phase 2. The intervention consists of a clinic-based component and a take-home component that will be tested in combination (e.g., one clinic-based component with one take-home component). The priming image will appear on all components.

Priming Image:

All components will include the priming image of a Baobab tree (the "tree of life"), a positive image known by residents in Shinyanga where the trees are numerous. It is immediately recognizable, associated with health and medicinal properties, and regarded as a symbol of life and positivity in a landscape where little else can thrive. This image was paired with the saying "*Together we can hug the Baobab tree*", a play on a widely known Tanzanian idiom about working together to achieve a goal. The saying was intended to convey the support available to patients in the clinic from each other, the staff, and the community. The image can be seen in **Appendix 1**.

Clinic-Based Component:

An interactive poster at the clinic. Patients who pick up their medication on time or achieve other accomplishments (e.g., three consecutive on-time medication pick-ups) will be given a small colored sticker to place on an interactive poster that is publicly displayed at the clinic. For example, the poster may be in the shape of a tree and the stickers are the "leaves." In this activity, the patient feels positive emotions about their accomplishment and experiences pride as others see him/her place their sticker on the wall. Also, the growing accumulation of stickers provides important feedback about how many patients at the clinic are adherent (e.g., being adherent is the normative behavior). One of the posters can be seen in **Appendix 2**.

Take-Home Components:

1. **A plastic take-home pillbox (Clinic A).** We will provide patients a small take-home plastic pill case. We have learned of elaborate systems patients develop to hide their pill bottles, which often results in them missing doses of their medication when they are away from home. We hypothesize that having a simple pill case (<$0.80) may alleviate some of this stress and improve adherence. This pill case will have the Baobab logo (**Appendix 3**).
2. **A take-home calendar (Clinic B).** Patients will be given a 1-page calendar with images of strength and happiness using stock photos that tested well in focus group discussions (see prototype in the Appendix). It will include the logo of the Baobab tree (**Appendix 4**).

B. Pilot Study: In this evaluation phase of the study, UC Berkeley researchers will not have direct contact with patients. The intervention will be implemented by health facility staff and all data collection will be conducted by the local research team. Participating health facilities will implement the finalized intervention(s) for the first two weeks of each month for 6 months. This will mean installing the clinic-based component described above plus one of the take-home components (e.g., calendar or pill box). To evaluate the intervention, we will use existing medical record data to compare the adherence levels of patients who were exposed to the intervention to a similar group of patients who were unexposed.

Specifically, the research team will utilize existing medical and pharmacy records from selected clinics. Clinic staff will acquire these data as part of routine medical visits for HIV patients at the care and treatment clinics. Because patients typically follow 30 or 60-day schedules, we will be able to use these existing data to construct two retrospective cohorts of patients who are exposed and unexposed to the intervention. We will randomly sample PLHIV from clinic registers who had visits during the intervention weeks (i.e., they received a take-home component and potentially earned a sticker on their first visit) and comparison weeks. This will allow us to determine whether there is any preliminary evidence of the intervention's success (to be evaluated in a future, adequately powered impact study).

Because this is a pilot study, we have not conducted formal power calculations to determine an ideal sample size. However, we plan to review at least 200 pharmacy records of patients exposed to the intervention and 200 records of those unexposed to the intervention. We plan to randomly sample more than 400 patients in order to account for missing records, ineligible patients, and other data issues that would preclude inclusion in the study. All data analyses will be weighted to account for the sampling design.

Primary Outcomes:

1. Retention in care 6 months after baseline, defined as a visit between 150 and 210 days after the baseline visit (6 months +/- 30 days).

2. Medication possession ratio (MPR) ≥95%. MPR is the proportion of time an individual is in possession of >1 ARV or prescription for ARV[17]. MPR is computed as the number of days ARVs are prescribed or dispensed divided by the number of days in the interval, and has been shown to be associated with short-term virologic outcomes.[18] We will determine the proportion of patients with MRP ≥95%. Due to budgetary constraints, we cannot test viral suppression of the study's subjects, but MPR is a suitable alternative that is used in many adherence studies.

Secondary Outcomes:
Secondary outcomes were "appointment adherence," the proportion of scheduled visits that are completed during the 6-month observation period, and MPR on a continuous scale.

<u>C. Patient and provider satisfaction surveys</u> will be conducted at baseline and endline to determine intervention feasibility and acceptability (i.e., staff support for treatment and life goals, overall satisfaction with care received at the clinic, and experience with intervention components.) The surveys will be completely anonymous and not linked to the population sampled for medical records.

For the patient surveys, members of the research team will approach patients who are waiting for care at the adult (>=18 years) HIV clinic or have completed their visit and ask if s/he would be willing to answer a few brief questions about their experience at the clinic. If so, they will move to a private space outdoors or inside the clinic where they can talk with our research staff member. We will choose a quiet section of the clinic or outside (most clinics are "open air" and partially outside) for the satisfaction surveys and try to provide as much privacy to participants as possible when asking the questions.

For the provider surveys, members of the research team will approach health care workers at the beginning of the day and tell them about the survey. We will tell them they can do the survey now or later in the day, whenever they have time to complete the survey. We will follow the same procedures for ensuring privacy (e.g., we will use a locked, private office or outdoor). We will follow an IRB-

approved recruitment and consent script. These anonymous data will be entered directly into Microsoft Excel data collection form on a study laptop. We anticipate conducting ~ 200 rapid interviews with patients at baseline, and ~200 at completion of the intervention period. Similarly, we will interview no more than 30 health care workers at baseline, and 30 at endline. In total, we will have a total of 460 satisfaction interviews.

INSTITUTIONAL ETHICAL CLEARANCE & ETHICAL CONSIDERATION

The research team has extensive training in the protection of human subjects and research ethics. In addition to receiving approval from the National Institute of Medical Research in Tanzania this research proposal has been approved by the Committee for the Protection of Human Subjects at the University of California, Berkeley (http://cphs.berkeley.edu). Human subjects research will not begin until both approvals are in place.

The research team takes ethical issues very seriously and has considered them below for each phase of the study. Here we have concentrated on potential risks and discomforts to participants.

Phase 1: There is no risk of physical harm associated with this phase of the study. The primary risks of the research proposed in Phase 1 are psychological and the risk associated with loss of confidentiality. For example, during interviews, home visits, and FGDs, subjects might feel uncomfortable discussing their or their community's experience with HIV/AIDS, treatment regimens, or other topics related to their daily lives. However the interviews will only focus on areas that the subject raises as important, and subjects can always choose to not answer any of the questions. Participants may also feel uncomfortable with household observations, but research staff will visit only participants who volunteer for a household observation. The investigators will work with local leaders to understand the expectations around hospitality, visiting a home, etc. before conducting any household observations. There is a small risk that subjects participating in the photo collection activity will take pictures of other people without seeking their permission; however if this happens it is unlikely to result in any long-term anxiety or a breach of confidentiality. Research staff will instruct subjects not to take pictures of others without their consent, and we have included this in our informed consent form.

The FGDs share similar psychological risks as interviews, although participants will mostly be asked to react to example intervention ideas rather than answer personal questions about themselves. Nevertheless, participants may inadvertently or spontaneously divulge private information about themselves to researchers; therefore we will adhere to strict protocols for confidentiality during the study. Maintaining a safe environment is of utmost importance, and research staff will cease interviews, observations, and FGDs at any time at the request of the participant.

Phase 2: There are no physical or psychological risks to participants associated with this phase of the study. The biggest risk is a breach of data confidentiality. However, this risk is minimal as all data will be de-identified except for several elements of dates and will stored on a secure password protected server only accessible to study investigators. Because the intervention is delivered at the clinic level to all patients, is intended to be a subtle cue or display of information, involves minimal to no risk, and can be evaluated using de-identified existing data, informed consent of all participants would be impractical. We have been granted a waiver of written informed consent by both IRBs (per HHS federal regulations (45 CFR 46.116(c))).

LIMITATIONS

There are a few important limitations to this research:

1) We will measure ART adherence with the medication possession ratio, which is the best measure available given the time and resources available for this study, but it is not as strong as measuring viral suppression. However, using the medication possession ratio is the best possible adherence measure given the pilot nature of the study and our budget. If the intervention demonstrates a signal of potential effectiveness, we will seek funding for a larger impact evaluation where viral suppression is the primary outcome.
2) During the household observations, the researcher will encourage the subject to continue his/her daily routine without making exceptions for the researcher, however we recognize that the mere presence of an unfamiliar person may cause the subject to act differently. We will do our best to mitigate these effects and feel that we can collect valuable information from this research activity despite the potential bias.
3) We will only measure the effectiveness of the intervention for 6 months. Although this will shed light on the potential value of the intervention, we will not know whether the effect, if any, erodes over time.
4) Although our findings may be generalizable to PLHIV in low resource settings, given the nuanced ethnographic work that will be done to design the intervention, it might be possible that the results are only applicable to groups in Shinyanga or to other Tanzanians.
5) If the intervention is successful we do not have the resources to immediately scale-up the program to all PLHIV. However, we plan for the intervention to be easy to implement and inexpensive, so community based organizations may be able to implement the intervention without additional financial support.

DISSEMINATION OF RESEARCH RESULTS

Once our analysis is complete, we will immediately share the results with the Ministry of Health and Social Welfare in addition to the clinics that helped pilot the intervention. We will also communicate the results to the Bill and Melinda Gates Foundation in hopes of progressing to a second round of funding, which would allow for a larger impact evaluation of the program. Finally, we will disseminate our findings to the global scientific and policy community by publishing the analysis in a peer-reviewed journal, sharing the results at a conference, or pursuing another appropriate policy outlet. We may also informally document the interdisciplinary approach we took through policy briefs, blog posts, and other media.

Appendix 1: Logo

Baobab Tree Logo ("*Together we can hug the Baobab Tree*")

Appendix 2: Clinic Display

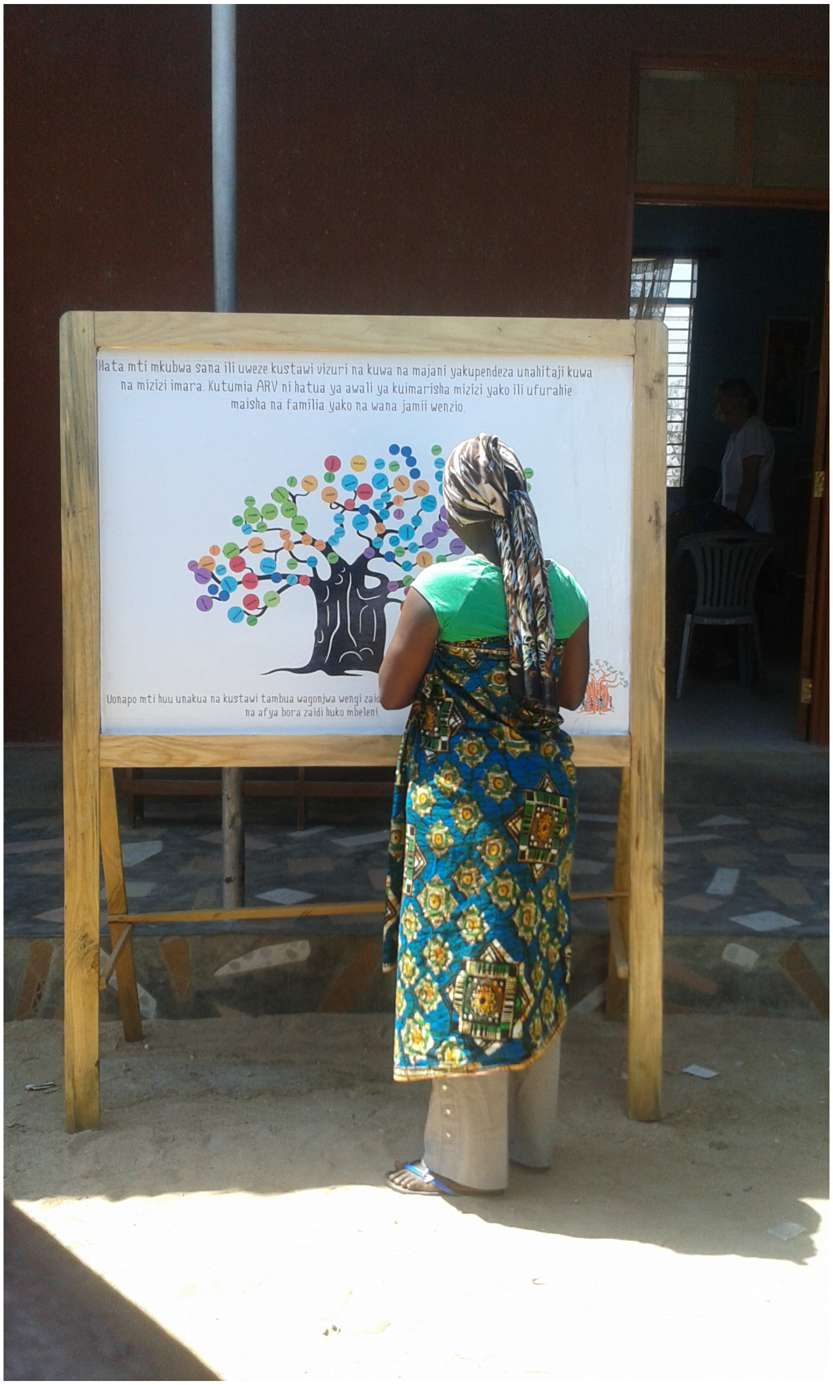

Appendix 3: Pillbox

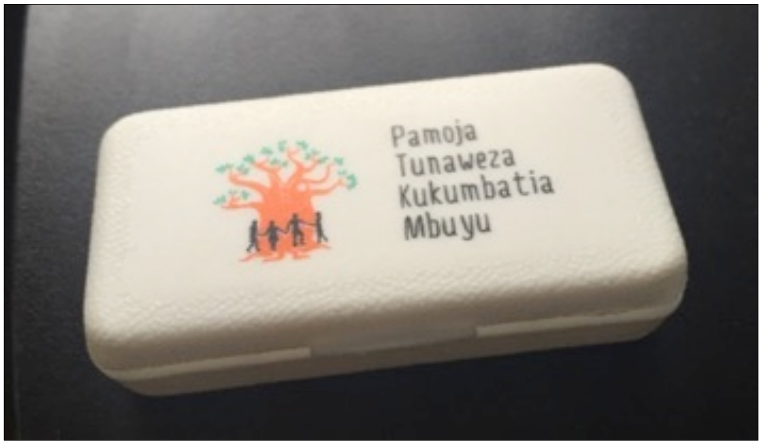

Appendix 4: Calendar Prototype

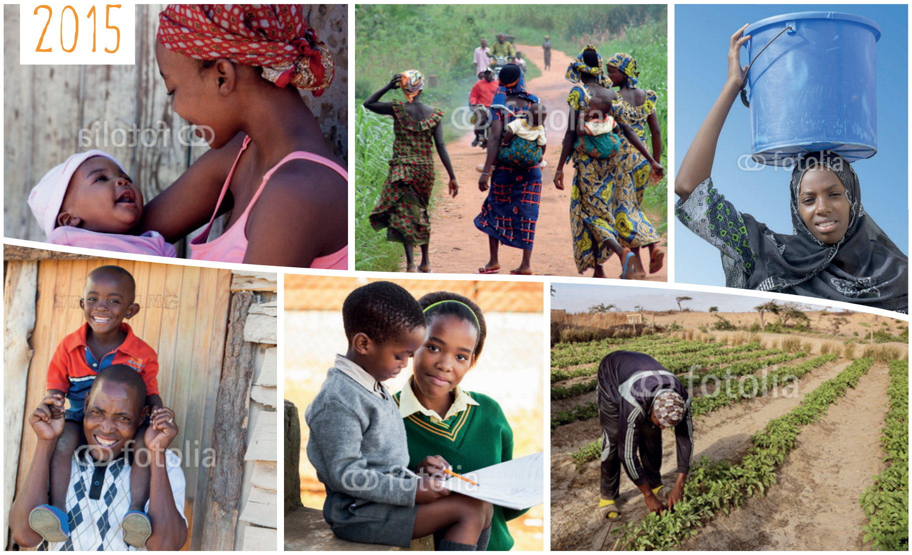

January

S	M	T	W	T	F	S
				1	2	3
4	5	6	7	8	9	10
11	12	13	14	15	16	17
18	19	20	21	22	23	24
25	26	27	28	29	30	31

February

S	M	T	W	T	F	S
1	2	3	4	5	6	7
8	9	10	11	12	13	14
15	16	17	18	19	20	21
22	23	24	25	26	27	28

March

S	M	T	W	T	F	S
1	2	3	4	5	6	7
8	9	10	11	12	13	14
15	16	17	18	19	20	21
22	23	24	25	26	27	28
29	30	31				

April

S	M	T	W	T	F	S
			1	2	3	4
5	6	7	8	9	10	11
12	13	14	15	16	17	18
19	20	21	22	23	24	25
26	27	28	29	30		

May

S	M	T	W	T	F	S
					1	2
3	4	5	6	7	8	9
10	11	12	13	14	15	16
17	18	19	20	21	22	23
24	25	26	27	28	29	30
31						

June

S	M	T	W	T	F	S
	1	2	3	4	5	6
7	8	9	10	11	12	13
14	15	16	17	18	19	20
21	22	23	24	25	26	27
28	29	30				

July

S	M	T	W	T	F	S
			1	2	3	4
5	6	7	8	9	10	11
12	13	14	15	16	17	18
19	20	21	22	23	24	25
26	27	28	29	30	31	

August

S	M	T	W	T	F	S
						1
2	3	4	5	6	7	8
9	10	11	12	13	14	15
16	17	18	19	20	21	22
23	24	25	26	27	28	29
30	31					

September

S	M	T	W	T	F	S
		1	2	3	4	5
6	7	8	9	10	11	12
13	14	15	16	17	18	19
20	21	22	23	24	25	26
27	28	29	30			

October

S	M	T	W	T	F	S
				1	2	3
4	5	6	7	8	9	10
11	12	13	14	15	16	17
18	19	20	21	22	23	24
25	26	27	28	29	30	31

November

S	M	T	W	T	F	S
1	2	3	4	5	6	7
8	9	10	11	12	13	14
15	16	17	18	19	20	21
22	23	24	25	26	27	28
29	30					

December

S	M	T	W	T	F	S
		1	2	3	4	5
6	7	8	9	10	11	12
13	14	15	16	17	18	19
20	21	22	23	24	25	26
27	28	29	30	31		

Pamoja Tunaweza Kukumbatia Mbuyu

REFERENCES

[1] UNAIDS: **Report on the Global AIDS Epidemic**. In. Geneva; 2013.
[2] Mills EJ, Nachega JB, Buchan I, Orbinski J, Attaran A, Singh S, Rachlis B, Wu P, Cooper C, Thabane L *et al*: **Adherence to antiretroviral therapy in sub-Saharan Africa and North America: a meta-analysis**. *Jama* 2006, **296**(6):679-690.
[3] Cohen MS, Chen YQ, McCauley M, Gamble T, Hosseinipour MC, Kumarasamy N, Hakim JG, Kumwenda J, Grinsztejn B, Pilotto JH *et al*: **Prevention of HIV-1 infection with early antiretroviral therapy**. *N Engl J Med* 2011, **365**(6):493-505.
[4] Chaiyachati KH, Ogbuoji O, Price M, Suthar AB, Negussie EK, Barnighausen T: **Interventions to improve adherence to antiretroviral therapy: a rapid systematic review**. *Aids* 2014, **28 Suppl 2**:S187-204.
[5] Bargh JA: **The Historical Origins of Priming as the Preparation of Behavioral Responses: Unconscious Carryover and Contextual Influences of Real-World Importance.** *Social Cognition* 2014, **32**: 209-224.
[6] Bargh JA, Chen M, Burrows L: **Automaticity of Social Behavior: Direct Effects of Trait Construct and Stereotype Activation on Action**. *Journal of Personality and Social Psychology* 1996, **71**(4): 230-244.
[7] Ibid
[8] Fitzsimons GM, Chartrand TL, Fitzsimons GJ: **Automatic Effects of Brand Exposure on Motivated Behavior: How Apple Makes You "Think Different"**. *J of Consumer Research* 2008, **35**:21-35. And Holland RW, Hendriks M, Aarts H: **Smells like clean spirit. Nonconscious effects of scent on cognition and behavior**. *Psychological science* 2005, **16**(9):689-693.
[9] Dupas P: **Do Teenagers Respond to HIV Risk Information? Evidence from a Field Experiment in Kenya**. In. Los Angeles: University of California; 2010.
[10] Grierson B: **What if Age is Nothing but a Mind-Set?** *New York Times* 22 Oct 2014. and Hsu L, Chung J, Langer E: **The Influence of Age-Related Cues on Health and Longevity**. *Perspectives on Psychological Science* 2010, **5**(6): 632-648.
[11] Dolan P, Hallsworth M, Halpern D, King D, Metcalfe R, Vlaev I. Influencing behaviour: the mindspace way. Journal of Economic Psychology. 2012;33(1):264-77.
[12] Martin S. 98% of HBR Readers Love This Article. Harvard Business Review. 2012.
[13] Hallsworth M, Chadborn T, Sallis A, Sanders M, Berry D, Greaves F, et al. Provision of social norm feedback to high prescribers of antibiotics in general practice: a pragmatic national randomised controlled trial. Lancet. 2016. doi: 10.1016/S0140-6736(16)00215-4. PubMed PMID: 26898856.
[14] Ehrhardt-Martinez K, Donnelly KA, Laitner JA: **Advanced Metering Initiatives and Residential Feedback Programs: A Meta-Review for Household Electricity-Saving Opportunities**. In: *Research Report E105.* Washington, D.C.: American Council for an Energy-Efficient Economy; 2010.
[15] Moreira MT, Smith LA, Foxcroft D: **Social norms interventions to reduce alcohol misuse in university or college students**. *Cochrane database of systematic reviews* 2009(3):CD006748.
[16] Ulin PR, Robinson ET, Tolley EE: **Qualitative Methods in Public Health: A Field Guide for Applied Research**. New York: John Wiley & Sons; 2004.
[17] McMahon JH, Jordan MR, Kelley K, Bertagnolio S, Hong SY, Wanke CA, Lewin SR, Elliott JH: **Pharmacy adherence measures to assess adherence to antiretroviral therapy: review of the literature and implications for treatment monitoring**. *Clin Infect Dis* 2011, **52**(4):493-506.
[18] McMahon et al (2011); and Messou E, Chaix ML, Gabillard D, Minga A, Losina E, Yapo V, Kouakou M, Danel C, Sloan C, Rouzioux C *et al*: **Association between medication possession ratio, virologic failure and drug resistance in HIV-1-infected adults on antiretroviral therapy in Cote d'Ivoire**. *J Acquir Immune Defic Syndr* 2011, **56**(4):356-364; and Goldman JD, Cantrell RA, Mulenga LB, Tambatamba BC, Reid SE, Levy JW, Limbada M, Taylor A, Saag MS, Vermund SH *et al*: **Simple adherence assessments to predict virologic failure among HIV-infected adults with discordant immunologic and clinical responses to antiretroviral therapy**. *AIDS Res Hum Retroviruses* 2008, **24**(8):1031-1035; and Hong S, Nachega J, Jerger L, Cohen S, Jonas A, Badi A, Oereko D, Trotter A, Wanke C, Jordan M: **Medication Possession Ratio Predictive of Short-term Virologic and Immunologic Response in Individuals Initiating ART: Namibia**. In: *19th Conference on Retroviruses and Opportunistic Infections.* Seattle; 2012.
