# Supplementary material for: Pilot study of a multi-pronged intervention using social norms and priming to improve adherence to antiretroviral therapy and retention in care among adults living with HIV in Tanzania
Source: PLoS One. 2017 May 9;12(5):e0177394. doi: 10.1371/journal.pone.0177394 (PMC5423659; doi:10.1371/journal.pone.0177394)
Supplement: S2 File — (PDF) [file pone.0177394.s006.pdf]

Interview Tools

Contents:

1. In-depth interview guide (Kiswahili)
2. In-depth interview guide (English)
3. Photo-Based Interview Guide (Kiswahili)
4. Photo-Based Interview Guide (English)
5. Patient Satisfaction Survey – Baseline (Kiswahili)
6. Patient Satisfaction Survey – Baseline (English)
7. Patient Satisfaction Survey – Endline (Kiswahili)
8. Patient Satisfaction Survey – Endline (English)

In-Depth Interview Guide (Kiswahili)

KUWAANDAA WAATHIRIKA WA VVU/UKIMWI KUTUMIA DAWA ZA ART TANZANIA: Mwongozo wa maswali ya mahojiano ya kina.

Maelekezo:

1) Maswali yalioandaliwa kwa kukazia na wino hapa chini yanatoa mchanganuo wa maswali. Vipengele vingine vinaelezea mada mbali mbali kwa madodoso Zaidi. Hakuna haja ya kuuliza kila kipengele ila tu kuzingatia maeneo ambayo wahojiwa wanakuwa na mengi Zaidi ya kuelezea.

2) Zingatia kuandika jinsia na umri(japo kwa kukisia) wa muhojiwa

Nieleze kuhusiana na muda uliokaa hapa kiliniki leo . Kipi kizuri na kipi kibaya kuhusiana na leo

- Ni nzuri au mbaya kuliko mara ya mwisho ulipokuja? Tofauti ni ipi?
- Unadhani madaktari na wauguzi wanahitaji wewe urudi tena kliniki? Kwanini?
- Unawapenda madkatri na wauguzi wa hapa? Wanaonyesha hali ya urafiki? Wanawaheshimu? Vipi kuhusu wafanyakazi wa chumba cha dawa?
- Umewahi kutazama matangazo yoyote hapa kliniki? Kama jibu ni NDIYO uliza kuhusu ujumbe mkuu katika matangazo au tangazo hilo. Uliza nini alikiona katika tangazo(ujumbe,picha, etc.)

Unadhani watu wengine wanazingatia vizuri matumizi ya ART? Unafikiri ni wangapi wanatumia dawa kila siku?

Nieleze uzoefu wako wa kutumia ART

- Kwa haraka haraka/ kukisia ni kwa muda gani umekua unatumia hizi dawa?
- KAMA MTU AMEANZA KUTUMIA DAWA NDANI YA MWAKA: ULIZA: nani alikueleza kuwa unahitaji kutumia ART? Mazungumzo hayo yalikuaje? Ulijisikiaje baada ya hapo?.
- Siku zote unatumia muda ule ule? Ni wakati gani?
- Je ni vigumu kukumbuka kutumia dawa kila siku? Je ni vigumu kukumbuka kurudi kuchukua dawa nyingine? Una mbinu yoyote inayokusaidia kukumbuka?
- Je imekua inakuwia vigumu mara zote au ilikuwa rahisi Zaidi ulipoanza kutumia dawa? Au ilikua vigumu Zaidi baadae? Kuna utofauti gani sasa na hapo mwanzo?
- Ulijua maudhi au madhara ya dawa yakutarajia?
- Je marafiki au familia huwa wanakukumbusha kutumia dawa? Wewe umewahi kuwakumbusha wengine?
- Je wauguzi au wahudumu wa kliniki huwa wanakupongeza au kukusifu kwa kukumbuka kutumia dawa ?huwa wanakupa ndondoo za namna ya kutumia dawa?umewahi kufuata ushauri wao? wamewahi kukugombeza kwa kutokutumia dawa?

- Umewahi kuhifadhi ART katika kihifadhio kingine ukiachilia mbali kopo lake ambalo ulipatiwa nalo dawa (toa mfano: namna anavyobeba akisafiri?)
- Unafikiri ART zina uthamani? Unadhani ni kitu muhimu au mtu yeyote tu anaweza kuzipata?

Hadi sasa saa moja yaweza ikawa imepita tayari. Kama saa moja imepita, sema kwamba unafurahia mazungumzo na kama unaweza kuuliza maswali machache Zaidi. Kama mgonjwa akikubali waweza kuendelea. Kama akikataa kuendelea na mazungumzo, sitisha mahojiano

Utajuaje kama mtu ana afya?

- Utajuaje kama mtu ana nguvu?

Nieleze kuhusiana na familia yako

- Una watoto, Ndugu au wapwa ambao unawahudumia? Kuna mtu mwingine yeyote unayemuhudumia?
- (kwa watoto tu) unadhani (watoto, ndugu wapwa) zako watakua wana hali gani katika kipindi cha mwaka kesho? Unatumaini nini kwao katika maisha yao ya baadae?

Unatarajia nini katika miezi sita ijayo? Kwanini?
(usiulize kama mtu amekutwa na majanga makubwa hivi karibuni kama vile kuharibiwa mazao yake na hali ya hewa)

Nani ana ushawishi katika maisha yako? Waweza kueleza namna ambavyo mtu huyo alikushawishi kufanya jambo lolote?

Hitimisho: asante sana kwa kutumia muda wako kuongea nasi. Una jambo lingine lolote ungependa kutueleza

In-Depth Interview Guide (English)

Priming HIV-Infected Patients to Adhere to Antiretroviral Therapy in Tanzania:
In-Depth Semi-Structured Patient Interview Guide

Instructions:

1) The bolded questions below outline main categories of questioning. The sub bullets detail topics for further probing. There is no need to ask every question, but rather to focus on the areas in which the respondent has the most to say.

2) Note down the respondent's rough age and gender.

Tell me about your time at the clinic today. What's been good or bad about it?

- Is it better or worse than the last time that you came? What's different?
- Do you think your doctor or nurse wants you to come back? Why?
- Do you like the doctors and nurses here? Are they friendly? Are they respectful? What about the staff at the pharmacy?
- Have you ever looked at any of the posters in the clinic? If YES, then ask them to repeat the main messages. Ask what was in the poster (text, image, etc.)

Do you think other patients at this clinic do a good job of taking their ART? How many of them do you think take it everyday?

Tell me about your experiences taking ART.

- Roughly how long have you been taking it?
- IF PERSON HAS INITIATED IN THE PAST YEAR: Who first explained to you that you needed to take ART? How was that conversation? How did you feel afterward?
- Do you always take it at the same time each day? When?
- Is it difficult to remember to take it everyday? Is it difficult to remember when to refill it? Do you have any tricks to help you remember?
- Has it always been difficult or was it easier when you first started taking the medication? Or was it harder then? What's different between now and then?
- Did you know what side effects to expect?
- Do friends or family ever remind you to take it? Do you ever remind others?
- Does the clinic staff members congratulate or praise you for remembering to take it? Do they ever give you tips on how to remember to take it? Have you ever tried their advice (ask if they belong to a community/peer support group)? Have they ever scolded you for not taking it?
- Do you ever store your ART in something other than the bottle it was given in? (give the example of when traveling)

- Do you think ART is valuable? Is it something significant or something that anyone can access?

At this point you may have reached one hour. If an hour has passed, say that you are enjoying the conversation and ask whether you may ask a few more questions. If the patient agrees, then continue. If they would like to stop, end the interview.

How do you know if a person is healthy?

- How do you know if a person is strong?

Tell me about your family.

- Do you have children, siblings, nieces or nephews that you help take care of? Do you take care of anyone else?
- (for children only) What do you think your (children, siblings, nieces, nephews) will be up to this time next year? What do you hope for them for their future?

What are you looking forward to in the next 6 months? Why? (don't ask if the person has recently experienced a major tragedy like weather destroying their crops)

Who in your life motivates or influences you? Can you describe a time when that person has motivated you to do something?

Concluding Remarks: Thank you so much for taking the time to speak with us today. Is there anything else that you'd like to tell me?

Photo-Based Interview Guide (Kiswahili)

KUWAANDAA WAATHIRIKA WA VVU/UKIMWI KUTUMIA DAWA ZA ART KWA USAHIHI

Maelekezo: Maswali yaliyoandaliwa kwa kukazia wino hapo chini yanatoa mchanganuo wa maswali. Vipengele vingine vinaelezea mada mbali mbali kwa ajili ya utafiti, hakuna haja ya kuuliza kila swali ila tu kwa kuzingatia maeneo ambayo wahojiwa wanakuwa na mengi ya kuzungumzia/kuelezea.
(Jambo la kuzingatiwa kwa mhojaji - Andika umri na jinsi ya mhojiwa)

Nieleze kuhusu picha ulizopiga

- Kwa nini umechagua kupiga picha watu hawa, mahali na vitu hivi?
- Nieleze kuhusu picha hizi(chagua picha ambazo zinaendana/zinazoleta mshangao)
    - Inakukumbusha hisia gani Zaidi?
    - Inakukumbusha nini Zaidi? Au wakati mwingine?
    - Unadhani kuwa inakukumbusha mambo mengine ya hisia za aina hii? Kwa nini hapana?
- Ni picha ipi unadhani kuwa inafanana/kufananishwa kwa uzoefu wako wa kupokea huduma za matibabu katika kliniki?
    - Unaweza ukanieleza Zaidi ni kwa nini ulipiga picha hii?
    - Unadhani inaweza kuwakumbusha wengine jambo fulani katika kliniki hii?
- Kuna kitu kingine Zaidi ungependa kupiga picha?
- Kama ungefenya kazi hii tena, unakuna kitu chochote unadhani kuwa usingekipiga picha tena?
- Kwa nini ulipenda kushiriki katika zoezi hili? Ulufahamu kwa hakika ulichotaka kupiga picha?

MAONI YA HITIMISHO: Tunakushukuru sana kwa kutumia mda wako kuzungumza nasi leo. Kuna jambo linguine la ziada kutueleza?

Photo-Based Interview Guide (English)

Priming HIV-Infected Patients to Adhere to Antiretroviral Therapy in Tanzania:
In-Depth Semi-Structured Patient Interview Guide, Photo Activity

Instructions: The bolded questions below outline main categories of questioning. The sub bullets detail topics for further probing. There is no need to ask every question, but rather to focus on the areas in which the respondent has the most to say.

(Note to Interviewer—note down respondent's age and sex.)

Tell me about the pictures you took.

- Why did you choose to take pictures of these people, places, and objects?
- Tell me about this picture (choose pictures that are most relevant or surprising).
  - What emotion does it remind you of most?
  - Does it always remind you of this? Or only sometimes?
  - Do you think it reminds others of the same emotion? Why or why not?
- Which of these pictures do you think is most related to your experience receiving care and treatment from the clinic?
  - Can you tell me more about why you took this picture?
  - Do you think this might remind others of the clinic as well?
- Is there anything else that you wished you could photograph?
- If you could do this again is there anything that you might not photograph again?
- What was it like to do this activity? Did you know right away what you wanted to photograph?

Concluding Remarks: Thank you so much for taking the time to speak with us today. Is there anything else that you'd like to tell me?

Patient Satisfaction Survey – Baseline

Baseline Patient Satisfaction Survey: Swahili Version (Used for interviews)

Recruitment & Informed Consent

"Ninaitwa_______ napenda kukukaribisha kushiriki katika utafiti unaofanywa na Wizara ya Afya na Ustawi wa Jamii pamoja na Chuo Kikuu cha California. Tungependa tukuulize maswali machache kuhusu uzoefu wako katika kliniki leo. Ni hiari kushiriki na pia utafiti hautakuwa na utambulisho wa mshiriki na utachukua takribani dakika 5, hautatozwa chochote kwa kukataa kujibu maswali. Je uko tayari kushiriki?"

KAMA HAPANA: → sitisha mjadala
KAMA NDIYO: → Je una umri wa miaka isiyopungua 18?

> **KAMA HAPANA**: → sitisha mjadala
> KAMA NDIYO:→ elekea sehemu yenye ukimya na usiri kisha anza mahojiano

Survey Questions

1. Je wahudumu wa afya hapa yaani madaktari, manesi na mtu wa chumba cha dawa wanakuhimizaje ili ufikie malengo yako ya matibabu?

1: Hawanihimizi   2: wananihimiza   3: wananihimiza sana

2**. Msaada wa wahudumu wa afya hapa yaani madaktari, manesi na mtu wa chumba cha dawa ukoje katika kukusaidia kufikisha malengo yako ya maisha kama vile ndoa, kuanzisha familia au kurejea tena kazini?**

1: Hawana msaada   2: wana msaada   3: wana msaada sana

3. Ni kwa kiwango gani unakubaliana au kutokukubaliana na sentensi ifuatayo: Ninafurahia kuwepo kliniki ikijumuisha na muda ninaokaa katika eneo la kusubiri huduma.

1: Sikubaliani kabisa
2: Sikubaliani
3: Nakubali
4: Nakubali sana

4. Je una swali lolote ambalo halina majibu na hukumuuliza muhudumu wa afya leo? Hata kama swali hilo halihusu mambo ya UKIMWI?

1. Ndio   2. Hapana

5. Je kuna maelekezo yoyote uliyopatiwa na mtoa dawa leo?

1. Ndio 2. Hapana

6. **Kama jibu ni 'NDIO' kwa swali namba 5 hapo juu, Je ni kwa kiwango kipi uliyaelewa maelekezo uliyopewa na mtoa dawa?**

1. Sikuelewa alichonieleza
2. Nilielewa baadhi tu ya aliyonieleza
3. Nilielewa yote aliyonieleza

7. Matarajio yako ya kurudi kliniki katika siku uliyopangiwa yapoje?. Waweza kuwa muwazi tu, mahojiano haya ni siri.

1. Hakika nitarejea katika siku niliyopangiwa kurudi
2. Sina hakika kama nitarejea au sitarejea katika siku niliyopangiwa kurudi
3. Yawezekana nisirejee katika siku niliyopangiwa kurudi

8. Matarajio ya kutumia matibabu yako yote ya ART kama yalivyopendekezwa mwezi huu yakoje?

1. Hakika nitatumia matibabu yangu yote kwa mwezi huu unaokuja
2. Sina hakika kama nitatumia au sitatumia matibabu yangu yote kwa mwezi huu unaokuja
3. Yawezekana nisitumie matibabu yangu yote kwa mwezi huu unaokuja

9. Kwa maoni yako, je uwezekano wa wagonjwa wengine katika kliniki hii kutumia matibabu yao yote ya ART kama yalivyopendekezwa mwezi huu yakoje?

1. Wagonjwa wengi watatumia matibabu yao yote kwa mwezi huu unaokuja
2. Baadhi ya wagonjwa watatumia matibabu yao yote kwa mwezi huu unaokuja
3. Wagonjwa wachache watatumia matibabu yao yote kwa mwezi huu unaokuja
4. Sina hakika ni wangapi watatumia matibabu yao yote kwa mwezi huu unaokuja

10. Ni kwa kiwango gani unakubaliana na sentensi ifuatayo: wagonjwa wengine hapa kliniki wana msaada kwangu.

1. Sikubaliani kabisa
2. Sikubali
3. Nakubali
4. Nakubali sana

11. Kwa ujumla unaridhishwaje na huduma na matibabu unayoyapata katika kliniki hii?

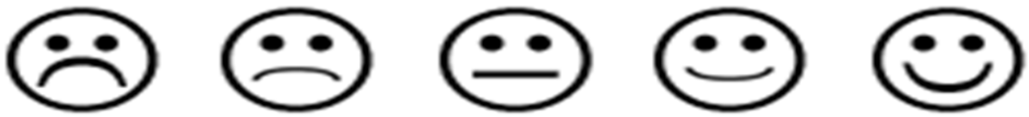

1: siridhiki kabisa	2: siridhiki	3: Si kwamba Naridhika au siridhiki	4: Naridhika	5:Naridhika sana

12.Una umri gani katika miaka?

13.Mhojiwa ni : 1: Mwanamme / 2: Mwanamke

Baseline Patient Satisfaction Survey: English Version

Recruitment & Informed Consent

"My name is ____ and I'd like to invite you to participate in a research study conducted by the Ministry of Health and Social Work and the University of California. We would like to ask you a few questions about your experience at the clinic today. This is a voluntary and anonymous survey that will take about 5 minutes, and there is no penalty for not wanting to answer the questions. Are you willing to participate?"

IF NO: → End discussion.
IF YES: → "Are you at least 18 years of age?"

IF NO: → End discussion.
IF YES: → Move to a quiet and private place, begin survey.

Survey Questions

1. How supportive are the clinic staff, including doctors, nurses, and pharmacists, in helping you meet your treatment goals?

1: Not supportive   2: Supportive   3: Very supportive

2. How supportive are the clinic staff, including doctors, nurses, and pharmacists, in supporting you to meet your life goals, such as marriage, having a family, and returning to work?

1: Not supportive   2: Supportive   3: Very supportive

3. How much do you agree or disagree with the following statement: I enjoy being at the clinic, including the time I spend in the waiting area.

1: Strongly disagree
2: Disagree
3: Agree
4: Strongly agree

4. Do you have any unanswered questions that you did not ask your health care providers today? Even if those questions are not about HIV infection?

1: Yes   2: No

5. Have you been given any instructions by the Pharmacist today?

1: Yes 2: No

6. If the answer to Question 5 above is 'YES', how well did you understand the instructions given to you by the pharmacist?

1. I didn't understand what s/he told me
2. I understood some of what s/he told me
3. I understood all of what s/he told me

7. How likely are you to return to the clinic on the day of your next scheduled appointment? It's OK to be honest, this survey is anonymous.

1. I will definitely return for my next scheduled appointment.
2. I am not sure whether or not I will return for my next scheduled appointment.
3. I will probably not return for my next scheduled appointment.

8. How likely are you to take all of your recommended ART treatment this month?

1. I will definitely take all of my treatment this next month.
2. I am not sure whether or not I will take all of my treatment this next month.
3. I am not likely to take all of my treatment this next month.

9. In your opinion, how likely is it that other patients in this clinic will take all of their recommended ART treatment this month?

1. Most patients will take all of their treatment this next month.
2. Some patients will take all of their treatment this next month.
3. Few patients will take all of their treatment this next month.
4. I am not sure if patients will take all of their treatment this next month

10. To what extent do you agree with the following statement: The other patients at the clinic support me.

1. Strongly disagree
2. Disagree
3. Agree
4. Strongly agree

11. Overall how satisfied are you with the services and care you receive at this clinic?

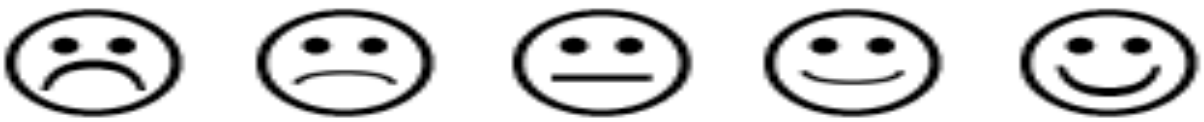

1: Very unsatisfied	2: Unsatisfied	3: Neither satisfied nor dissatisfied	4: Satisfied	5: Very satisfied

12. What is your age in years?

13. Respondent is: **1:** Male / 2: Female

Patient Satisfaction Survey - Endline

Endline Patient Satisfaction Survey: Swahili Version (Used for interviews)

Recruitment and Informed Consent

"Ninaitwa_______ napenda kukukaribisha kushiriki katika utafiti unaofanywa na Wizara ya Afya na Ustawi wa Jamii pamoja na Chuo Kikuu cha California. Tungependa tukuulize maswali machache kuhusu uzoefu wako katika kliniki leo. Ni hiari kushiriki na pia utafiti hautakuwa na utambulisho wa mshiriki na utachukua takribani dakika 5, hautatozwa chochote kwa kukataa kujibu maswali. Je uko tayari kushiriki?"

KAMA HAPANA: → sitisha mjadala
KAMA NDIYO: → Je una umri wa miaka isiyopungua 18?

> **KAMA HAPANA**: → sitisha mjadala
> KAMA NDIYO:→ elekea sehemu yenye ukimya na usiri kisha anza mahojiano

Survey Questions

1. Je wahudumu wa afya hapa yaani madaktari, manesi na mtu wa chumba cha dawa wanakuhimizaje ili ufikie malengo yako ya matibabu?

1: Hawanihimizi 2: wananihimiza 3: wananihimiza sana

2**. Msaada wa wahudumu wa afya hapa yaani madaktari, manesi na mtu wa chumba cha dawa ukoje katika kukusaidia kufikisha malengo yako ya maisha kama vile ndoa, kuanzisha familia au kurejea tena kazini?**

1: Hawana msaada 2: wana msaada 3: wana msaada sana

3. Ni kwa kiwango gani unakubaliana au kutokukubaliana na sentensi ifuatayo: Ninafurahia kuwepo kliniki ikijumuisha na muda ninaokaa katika eneo la kusubiri huduma.

1: Sikubaliani kabisa
2: Sikubaliani
3: Nakubali
4: Nakubali sana

4. **Je una swali lolote ambalo halina majibu na hukumuuliza muhudumu wa afya leo? Hata kama swali hilo halihusu mambo ya UKIMWI?**

1. Ndio 2. Hapana

5. Je kuna maelekezo yoyote uliyopatiwa na mtoa dawa leo?

1. Ndio 2. Hapana

6. **Kama jibu ni 'NDIO' kwa swali namba 5 hapo juu, Je ni kwa kiwango kipi uliyaelewa maelekezo uliyopewa na mtoa dawa?**

1. Sikuelewa alichonieleza
2. Nilielewa baadhi tu ya aliyonieleza
3. Nilielewa yote aliyonieleza

7. Matarajio yako ya kurudi kliniki katika siku uliyopangiwa yapoje?. Waweza kuwa muwazi tu, mahojiano haya ni siri.

1. Hakika nitarejea katika siku niliyopangiwa kurudi
2. Sina hakika kama nitarejea au sitarejea katika siku niliyopangiwa kurudi
3. Yawezekana nisirejee katika siku niliyopangiwa kurudi

8. Matarajio ya kutumia matibabu yako yote ya ART kama yalivyopendekezwa mwezi huu yakoje?

1. Hakika nitatumia matibabu yangu yote kwa mwezi huu unaokuja
2. Sina hakika kama nitatumia au sitatumia matibabu yangu yote kwa mwezi huu unaokuja
3. Yawezekana nisitumie matibabu yangu yote kwa mwezi huu unaokuja

9. Kwa maoni yako, je uwezekano wa wagonjwa wengine katika kliniki hii kutumia matibabu yao yote ya ART kama yalivyopendekezwa mwezi huu yakoje?

1. Wagonjwa wengi watatumia matibabu yao yote kwa mwezi huu unaokuja
2. Baadhi ya wagonjwa watatumia matibabu yao yote kwa mwezi huu unaokuja
3. Wagonjwa wachache watatumia matibabu yao yote kwa mwezi huu unaokuja
4. Sina hakika ni wangapi watatumia matibabu yao yote kwa mwezi huu unaokuja

10. Ni kwa kiwango gani unakubaliana na sentensi ifuatayo: wagonjwa wengine hapa kliniki wana msaada kwangu.

1. Sikubaliani kabisa
2. Sikubali
3. Nakubali
4. Nakubali sana

11.Kwa ujumla unaridhishwaje na huduma na matibabu unayoyapata katika kliniki hii?

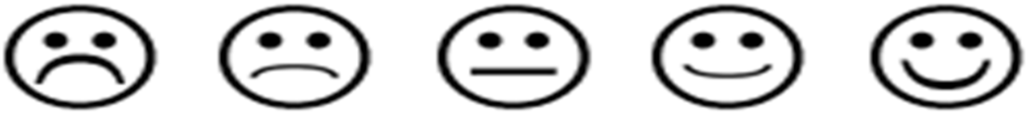

1: siridhiki kabisa	2: siridhiki	3: Si kwamba Naridhika au siridhiki	4: Naridhika	5:Naridhika sana

12. (CLINIC B ONLY) umewahi kupata chakula hapa kliniki katika kipindi cha miezi sita iliyopita?

1. Ndio
0. Hapana → **RUKIA SWALI NAMBA 14**

13. Kwanini ulipata hiko chakula

1. Kwa kuhudhuria kliniki kama ilivyopangwa
2. Sifahamu
3. Sababu nyingine; ainisha______________________

14. Katika kipindi cha miezi 6 iliyopita umewahi kuona bango kubwa la/ubao mkubwa wa mbao lenye vibandiko ya rangi mbalimbali hapa kliniki?

1. Ndiyo
1. Hapana→ **RUKIA SWALI NAMBA 18**

15.Je wewe umewahi kupata nafasi ya kubandika kibandiko katika bango/ubao huo?

1. Ndiyo
0. Hapana→ **RUKIA SWALI NAMBA 18**

16. Ulipopata kibandiko, ulijisikiaje?

____

17. Ni kwanini ulipata nafasi ya kubandika kibandiko katika ubao huo?

1. (jibu sahihi… kuhudhuria kliniki kama ilivyopangwa, kuwa mfuasi mzuri)
2. Sifahamu
3. Nimesahau
4. (jibu lisilo sahihi)

18. Katika kipindi cha miezi 6 iliyopita ulipata (kimkebe cheupe/kalenda) kutoka kliniki?

1. Ndiyo
0. Hapana→ **RUKIA SWALI NAMBA 22**

19. Ni mara ngapi katika kipindi cha miezi 6 iliyopita ulitumia kimkebe/ kalenda

1. Kila siku
2. Mara moja
3. Sijawahi kutumia kabisa
4. Nyinginezo (ainisha)________

20. (CLINIC A) ulitumiaje kalenda (zungushia majibu yote husika)

1. Kama pambo
2. Kufuatilia mahudhurio yangu na mambo mengine
3. Niliigawa
4. Matumizi mengine: ainisha________________________________

20. (CLINIC B) Ulitumiaje mkebe (zungushia majibu yote husika)

1. Kuhifadhia chakula, vitu binafsi mbalimbali
2. Nilikigawa
3. Nilihifadhia vidonge
4. Matumizi mengine; ainisha____________________________________

21. Je kuna ujumbe wowote au taswira yoyote unayoweza kuifikiria ukifikiri kuhusu (kimkebe/kalenda)?

____

____

22. Una umri gani katika miaka?____________

23. Mhojiwa ni : 1: Mwanamume / 2: Mwanamke

Endline Patient Satisfaction Survey: English Version

Recruitment & Informed Consent

"My name is _____ and I'd like to invite you to participate in a research study conducted by the Ministry of Health and Social Work and the University of California. We would like to ask you a few questions about your experience at the clinic today. This is a voluntary and anonymous survey that will take about 5 minutes, and there is no penalty for not wanting to answer the questions. Are you willing to participate?"

IF NO: → End discussion.
IF YES: → "Are you at least 18 years of age?"

IF NO: → End discussion.
IF YES: → Move to a quiet and private place, begin survey.

Survey Questions

1. How supportive are the clinic staff, including doctors, nurses, and pharmacists, in helping you meet your treatment goals?

1: Not supportive   2: Supportive   3: Very supportive

2. How supportive are the clinic staff, including doctors, nurses, and pharmacists, in supporting you to meet your life goals, such as marriage, having a family, and returning to work?

1: Not supportive   2: Supportive   3: Very suppportive

3. How much do you agree or disagree with the following statement: I enjoy being at the clinic, including the time I spend in the waiting area.

1: Strongly disagree
2: Disagree
3: Agree
4: Strongly agree

4. Do you have any unanswered questions that you did not ask your health care providers today? Even if those questions are not about HIV infection?

1: Yes   2: No

5. Have you been given any instructions by the Pharmacist today?

1: Yes 2: No

6. If the answer to Question 5 above is 'YES', how well did you understand the instructions given to you by the pharmacist?

1. I didn't understand what s/he told me
2. I understood some of what s/he told me
3. I understood all of what s/he told me

7. How likely are you to return to the clinic on the day of your next scheduled appointment? It's OK to be honest, this survey is anonymous.

1. I will definitely return for my next scheduled appointment.
2. I am not sure whether or not I will return for my next scheduled appointment.
3. I will probably not return for my next scheduled appointment.

8. How likely are you to take all of your recommended ART treatment this month?

1. I will definitely take all of my treatment this next month.
2. I am not sure whether or not I will take all of my treatment this next month.
3. I am not likely to take all of my treatment this next month.

9. In your opinion, how likely is it that other patients in this clinic will take all of their recommended ART treatment this month?

1. Most patients will take all of their treatment this next month.
2. Some patients will take all of their treatment this next month.
3. Few patients will take all of their treatment this next month.
4. I am not sure of the patients who will take all of their treatment this next month

10. To what extent do you agree with the following statement: The other patients at the clinic support me.

1. Strongly disagree
2. Disagree
3. Agree
4. Strongly agree

11. Overall how satisfied are you with the services and care you receive at this clinic?

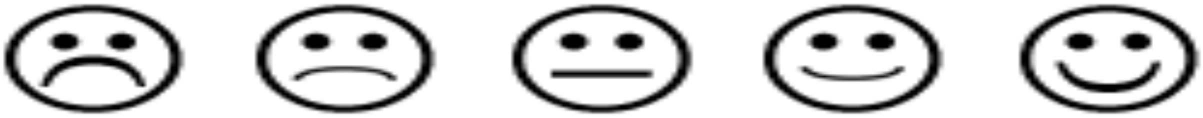

1: Very unsatisfied 2: Unsatisfied 3: Neither satisfied 4: Satisfied 5: Very
nor dissatisfied satisfied

12. [CLINIC B ONLY] Did you receive a free lunch at the clinic in the past six months?

1. Yes
0. No → SKIP to QUESTION 14

13. Why did you receive a free lunch?

1. Attended scheduled visit date
2. I don't know
3. Other reason: specify________________________

14. In the past 6 months, did you ever see a large poster at the clinic with multiple colored stickers?

1. Yes
0. No → SKIP TO QUESTION 18

15. Did you yourself ever get to put a sticker on the poster?

1. Yes
0. No → SKIP TO QUESTION 18

16. When you earned a sticker, how did you feel?

____

17. Why did you get to put a sticker on the poster?

1. (mostly correct answer...keeping appointments, being adherent)
2. I don't know
3. I don't remember
4. (incorrect answer)

18. In the past 6 months, did you receive a [pill box/calendar] from the clinic?

1. Yes
0. No → SKIP TO QUESTION 22

19. How often in the last 6 months did you [use the pillbox/look at the calendar]?

1. Daily
2. Once
3. Never
4. Others (specify)___________

20. [CLINIC A] How did you use the calendar? (Circle all that apply)

1. As decoration
2. To keep track of my appointments or other commitments
3. Gave it away
4. Used for another purpose: specify_____________

20. [CLINIC B] How did you use the pill box? (Circle all that apply)

1. Stored food, personal items, or other belongings in it
2. Gave it away
3. Stored my treatment in it
4. Used for another purpose: specify_____________

21. Was there a message or image that you think about when you think about the [pill box/calendar]?

____

22. What is your age in years?

23. Respondent is: **1:** Male / 2: Female
