# Supplementary material for: Pilot study of a multi-pronged intervention using social norms and priming to improve adherence to antiretroviral therapy and retention in care among adults living with HIV in Tanzania
Source: PLoS One. 2017 May 9;12(5):e0177394. doi: 10.1371/journal.pone.0177394 (PMC5423659; doi:10.1371/journal.pone.0177394)
Supplement: S1 Table — (DOCX) [file pone.0177394.s008.docx]

**S1 TABLE.** Results of sensitivity analyses to determine intervention effectiveness accounting for imperfect treatment exposure.

|  | **Mean (95% CI) at 6 months**^a^ | |  | **Between-group difference** | | | | | | | | |  |  |
| --- | --- | --- | --- | --- | --- | --- | --- | --- | --- | --- | --- | --- | --- | --- |
|  | Intervention (N=320) | Comparison (N=118) |  | Site-adjusted  logistic regression^d^ | | | |  | | Site-adjusted  IV analysis^e^ | | | | |
|  |  |  |  | Estimate (95% CI) | | | |  | | Estimate (95% CI) | | | | |
|  |  |  |  |  | | |  |  | |  | |  | | |
| **Retained in care**^b^ | 0.87 (0.85, 0.91) | 0.79 (0.73, 0.86) | | |  | 0.07 (0.01, 0.14)* | |  | 0.15 (0.01, 0.28)* | | | | |  |
|  |  |  | | |  |  |  |  |  | |  | | |  |
| **MPR ≥95%**^c^ | 0.70 (0.65, 0.74) | 0.59 (0.50, 0.67) | | |  | 0.08 (-0.01, 0.18) | |  | 0.16 (-0.02, 0.33) | | | | |  |
|  |  |  | | |  |  |  |  |  | |  | | |  |
|  |  |  |  |  | | |  |  | |  | |  | | |

*CI: confidence interval,* IV: instrumental variable

**p≤0.05*

1. *Weighted analyses to account for sampling strategy. These frequencies are repeated from Table 4 to facilitate data interpretation.*
2. *Whether the patient was in care at 6 months, defined as a visit between 150 and 210 days (6 months +/- 30 days). Excluding <1% of weighted sample who transferred before 6 months.*
3. *Medication possession ratio, the proportion of time an individual is in possession of ≥1 ART dose. Excluding 21% of weighted sample who were missing dispensing data for at least one recorded visit.*
4. *Predicted mean difference from a logistic regression model adjusted for site with treatment assignment according to the baseline visit week as the independent variable (consistent with the approach used in the primary analysis). P-value obtained from Wald test.*
5. *Results of two-stage least squares (2SLS) IV analysis using a linear probability model and adjustment for clinic. Treatment assignment according to the week of the baseline visit (intervention or control) was used as an instrument for the actual intervention exposure, which was defined as ≥50% of* *actual visits occurring on intervention weeks during the 6-month intervention period (binary).*
